# Heritable L1 retrotransposition in the mouse primordial germline and early embryo

**DOI:** 10.1101/gr.219022.116

**Published:** 2017-08

**Authors:** Sandra R. Richardson, Patricia Gerdes, Daniel J. Gerhardt, Francisco J. Sanchez-Luque, Gabriela-Oana Bodea, Martin Muñoz-Lopez, J. Samuel Jesuadian, Marie-Jeanne H.C. Kempen, Patricia E. Carreira, Jeffrey A. Jeddeloh, Jose L. Garcia-Perez, Haig H. Kazazian, Adam D. Ewing, Geoffrey J. Faulkner

**Affiliations:** 1 Mater Research Institute–University of Queensland, Woolloongabba QLD 4102, Australia;; 2 Invenra, Incorporated, Madison, Wisconsin 53719, USA;; 3 Department of Genomic Medicine, GENYO, Centre for Genomics and Oncological Research, Pfizer–University of Granada–Andalusian Regional Government, PTS Granada, 18016 Granada, Spain;; 4 Roche Sequencing Solutions, Incorporated, Madison, Wisconsin 53719, USA;; 5 Medical Research Council Human Genetics Unit, Institute of Genetics and Molecular Medicine, University of Edinburgh, Western General Hospital, Edinburgh EH4 2XU, United Kingdom;; 6 Institute of Genetic Medicine and Department of Pediatrics, Johns Hopkins University School of Medicine, Baltimore, Maryland 21205, USA;; 7 School of Biomedical Sciences,; 8 Queensland Brain Institute, University of Queensland, Brisbane QLD 4072, Australia

## Abstract

LINE-1 (L1) retrotransposons are a noted source of genetic diversity and disease in mammals. To expand its genomic footprint, L1 must mobilize in cells that will contribute their genetic material to subsequent generations. Heritable L1 insertions may therefore arise in germ cells and in pluripotent embryonic cells, prior to germline specification, yet the frequency and predominant developmental timing of such events remain unclear. Here, we applied mouse retrotransposon capture sequencing (mRC-seq) and whole-genome sequencing (WGS) to pedigrees of C57BL/6J animals, and uncovered an L1 insertion rate of ≥1 event per eight births. We traced heritable L1 insertions to pluripotent embryonic cells and, strikingly, to early primordial germ cells (PGCs). New L1 insertions bore structural hallmarks of target-site primed reverse transcription (TPRT) and mobilized efficiently in a cultured cell retrotransposition assay. Together, our results highlight the rate and evolutionary impact of heritable L1 retrotransposition and reveal retrotransposition-mediated genomic diversification as a fundamental property of pluripotent embryonic cells in vivo.

Long interspersed element 1 (LINE-1 or L1) is a mobile genetic element active in nearly all mammals (Furano 2000). L1 sequences mobilize via a copy-and-paste mechanism, termed retrotransposition (Moran et al. 1996), and comprise ∼18% of mouse DNA (Waterston et al. 2002). Each mouse genome harbors ∼3000 full-length retrotransposition-competent L1s (RC-L1s) belonging to three L1 subfamilies (T_F_, G_F_, and A) as well as nearly 600,000 L1 copies rendered immobile by 5′ truncation and the accumulation of internal mutations (Fanning 1983; Loeb et al. 1986; Padgett et al. 1988; Kingsmore et al. 1994; Takahara et al. 1996; DeBerardinis et al. 1998; Naas et al. 1998; Goodier et al. 2001; Waterston et al. 2002; Sookdeo et al. 2013). The ongoing production of new, heritable RC-L1 copies is therefore essential to preserve L1 mobility over evolutionary time. It follows that L1 mRNA and protein are expressed during germline and early embryonic development (Martin and Branciforte 1993; Branciforte and Martin 1994; Trelogan and Martin 1995; Garcia-Perez et al. 2007; Soper et al. 2008; Malki et al. 2014), and numerous host mechanisms regulate L1 activity during these stages (Yoder et al. 1997; Bourc'his and Bestor 2004; Watanabe et al. 2006, 2008; Aravin et al. 2008; Soper et al. 2008; Rowe et al. 2010; Zamudio and Bourc'his 2010; Wissing et al. 2011; Castro-Diaz et al. 2014; Crichton et al. 2014).

The developmental timing of only two heritable human L1 insertions has been elucidated; one event likely occurred in the female germline (Brouha et al. 2002), and the other occurred in a pluripotent embryonic cell and resulted in maternal somatic and germline mosaicism (van den Hurk et al. 2007). This result is consistent with reports of L1 retrotransposition in cultured human embryonic stem cells and induced pluripotent stem cells (iPSCs) (Garcia-Perez et al. 2007; Wissing et al. 2012; Klawitter et al. 2016), although a study of mouse iPSCs revealed little endogenous retroelement activity (Quinlan et al. 2011). Studies of transgenic L1 reporter animals have demonstrated retrotransposition in the germline (Ostertag et al. 2002; An et al. 2006) and in the early embryo (Kano et al. 2009). Surprisingly, in the latter study, transmission of engineered L1 insertions from mosaic parental animals to offspring was never observed, suggesting somatic but not germline contribution of insertion-harboring embryonic cells (Kano et al. 2009). Overall, the frequency and developmental timing of heritable L1 retrotransposition in vivo remain unclear. Here, we overcome the rarity of phenotype-causing endogenous retrotransposition events, and avoid the caveats of transgenic model systems, by adapting retrotransposon capture sequencing (RC-seq) (Baillie et al. 2011; Shukla et al. 2013) to detect the following presently active mouse endogenous retrotransposons: T_F_, G_F_, and A subfamily L1 elements, B1 and B2 SINEs, and IAP and ETn long terminal repeat (LTR) elements (mRC-seq) (Supplemental Fig. S1A; Mager and Stoye 2015; Richardson et al. 2015). We apply this technology to pedigrees of wild-type C57BL/6J mice to investigate the rate and developmental timing of heritable L1 insertions.

## Results

**Figure 1.**
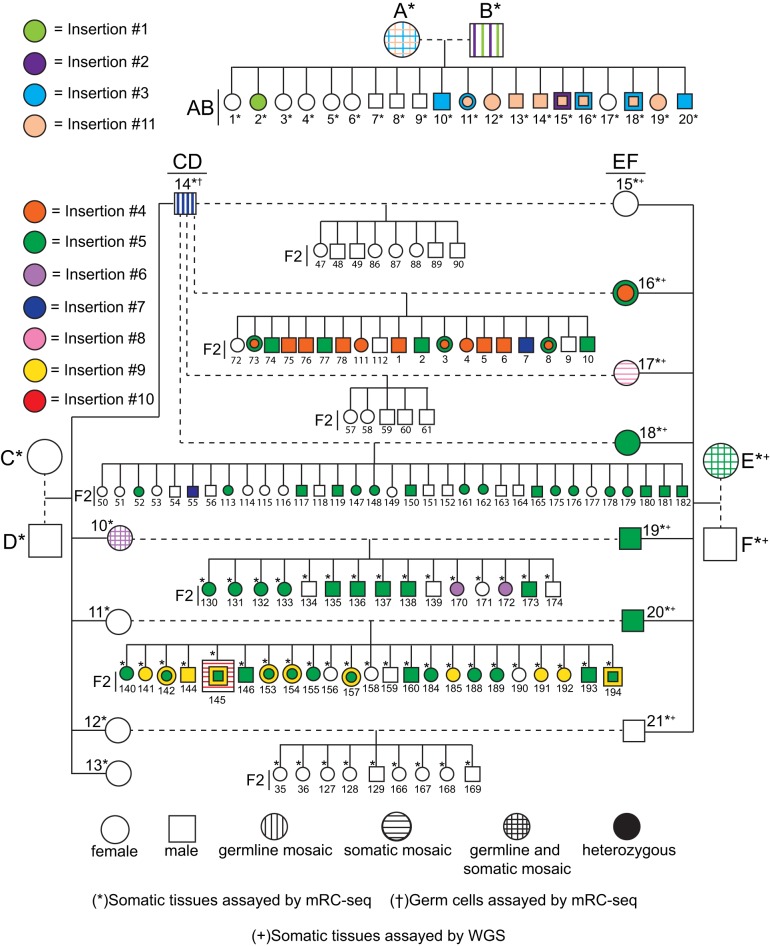
Origin and transmission of de novo L1 insertions in mouse pedigrees. Above, two-generation pedigree originating from parental mice SRA/SRB. Below, three-generation pedigrees originating from parental mice SRC/SRD and SRE/SRF. F1 animals are designated by the parental pair from which they arose (AB, CD, EF); F2 animals are so indicated. De novo L1 T_F_ insertions #1–#11 are color coded. In subsequent figures, schematics of each insertion are likewise color coded. With respect to each insertion, filled shapes indicate heterozygous animals, vertical hatching indicates germline mosaicism, horizontal hatching indicates somatic mosaicism, and vertical and horizontal hatching together indicates both somatic and germline mosaicism. Circles represent female animals; squares represent males. Dashed lines indicate matings. Animals for which mRC-seq was performed on somatic tissue gDNA are indicated with an asterisk; animals for which whole-genome sequencing (WGS) was performed on somatic tissue gDNA are indicated with a plus sign. For mouse SRCD14, mRC-seq was performed on somatic tissues as well as the germ cell fraction of the left and right testicles. Polymorphic retrotransposon insertions are not depicted in this figure.

We bred two- and three-generation pedigrees of C57BL/6J mice (Fig. 1), a strain known to accommodate recent L1 activity (Akagi et al. 2008), and used mRC-seq and whole-genome sequencing (WGS) to identify retrotransposon insertions absent from the C57BL/6J reference genome and not previously identified in an extensive analysis of polymorphic transposable element insertions across 13 commonly used inbred and four wild-derived mouse strains (Fig. 1; Supplemental Fig. S1; Methods; Nellaker et al. 2012). After sequencing analyses, we classified 28 L1 and short interspersed element (SINE) insertions present in at least one of the six “P-generation” animals (SRA/SRB, SRC/SRD, SRE/SRF) and absent from the C57BL/6J reference genome as polymorphic. Each “P-generation” animal harbored an overlapping subset of 17–19 polymorphic insertions; the specific polymorphic insertion content of each animal in the study can be found in Supplemental Table 2. Transmission of differentially present/absent polymorphic insertions was consistent with the known relationships among mice in our study (Fig. 1; Supplemental Table 2). Using data from mRC-seq reads and PCR validation followed by capillary sequencing, we discerned the complete structures of 17 polymorphic insertions (Supplemental Fig. S3; Supplemental Tables 2, 3), comprising 11 L1 T_F_ subfamily insertions, two B1 SINEs, and four B2 SINEs. Despite efficient detection of reference IAP and ETn retrotransposons (Supplemental Fig. S1C), we identified no polymorphic LTR insertions in our mice. This result may reflect differences in the activity levels of retrotransposon families among inbred mouse strains, revealed by numerous recent studies (Akagi et al. 2008, 2010; Quinlan et al. 2010; Keane et al. 2011; Li et al. 2012; Nellaker et al. 2012). For example, the majority of previously identified de novo IAP insertions arose on the IΔ1 subtype of the C3H/HeJ mouse strain (Maksakova et al. 2006).

We regarded insertions detected in one or more offspring but absent from the corresponding parents as potentially de novo (Supplemental Fig. S1B). Using PCR and capillary sequencing, we validated 11 de novo L1 insertions (Table 1; Supplemental Table 2). All 11 were T_F_ subfamily elements, consistent with previous reports of disease-causing L1 insertions in mice wherein all insertions for which sufficient L1 sequence was present for subfamily distinction were identified as T_F_ elements (Kingsmore et al. 1994; Mulhardt et al. 1994; Kohrman et al. 1996; Takahara et al. 1996; Perou et al. 1997; Naas et al. 1998; Yajima et al. 1999; Cunliffe et al. 2001). De novo L1 insertions bore hallmarks of L1 retrotransposition by target-primed reverse transcription (TPRT), including insertion at sequences resembling the L1 endonuclease cleavage motif (5′-TTTT/AA-3′), the presence of 13- to 17-bp target-site duplications (TSDs), and 3′ poly(A) tracts (Table 1; Supplemental Figs. S1E, S4; Singer et al. 1983; Scott et al. 1987; Luan et al. 1993; Moran et al. 1996; Jurka 1997). The average GC content of de novo L1 insertion sites within a 50-bp and 20-kb window of the endonuclease cleavage position was 30% and 38%, respectively, consistent with a previously described preference of L1 for AT-rich regions (Supplemental Table 4; Szak et al. 2002; Boissinot et al. 2004; Gasior et al. 2007). One insertion (insertion #5) (Fig. 3A, below) landed within the gene *Ano4,* which encodes a brain-expressed transmembrane protein of unknown function (Picollo et al. 2015; Petryszak et al. 2016)*.* Consistent with the observation that intronic L1 insertions in antisense orientation have little impact on RNA polymerase processivity (Han et al. 2004), we did not observe a decrease in *Ano4* mRNA levels in forebrain of animals heterozygous for this insertion (Supplemental Fig. S1G). Insertion #5 also occurred within an intronic L1mA5 element, and two additional intergenic insertions landed within existing L1 repeats (Supplemental Table 4).

**Table 1.**
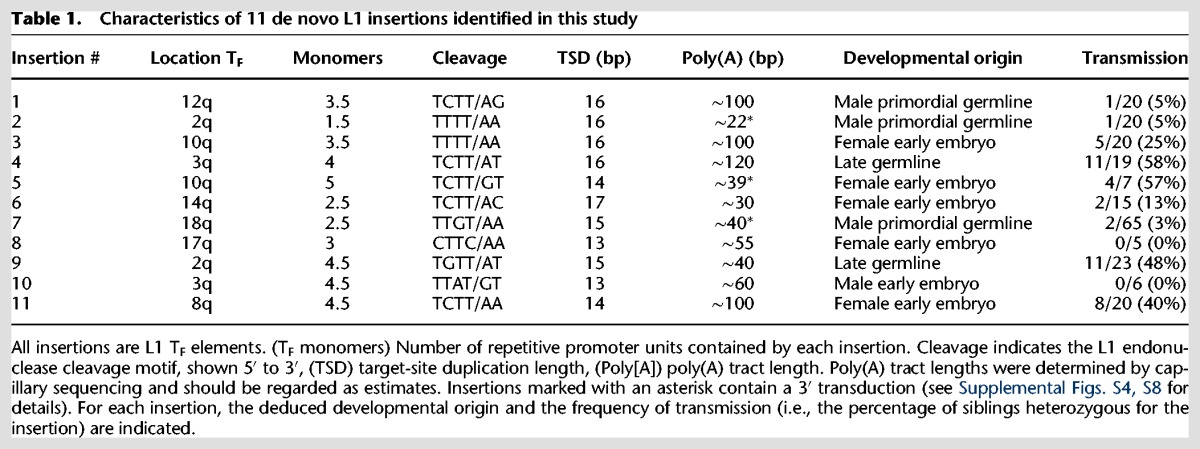
Characteristics of 11 de novo L1 insertions identified in this study

All 11 de novo insertions were 5′ detected by mRC-seq and thus full-length (containing ≥1 T_F_ monomer), reflecting depletion of mouse L1 3′ termini observed during Illumina sequencing (Supplemental Fig. S1D; Supplemental Tables 1, 2), possibly due to the GC-rich nature of the mouse L1 3′ end sequence (Chambers et al. 2015). Furthermore, the de novo L1 insertions had relatively long poly(A) tracts (average ∼64 bp), reducing the likelihood that L1 3′ end sequence and flanking genomic DNA would be captured in a single sequencing read. However, 20–30× WGS, which, in principle, could allow detection of the 5′ junctions of 5′ truncated L1 insertions, applied to nine mouse genomes (SRE, SRF, and offspring SREF15-21) uncovered no 5′ truncated de novo L1 insertions (Fig. 1; Supplemental Tables 1, 2), and a previous analysis suggested that T_F_ L1s undergo 5′ truncation less frequently than other L1 elements (Hardies et al. 2000). Complete internal sequencing of nine de novo L1 insertions revealed intact ORFs and the absence of mutations in critical functional domain residues (Supplemental Fig. S2; Furano 2000).

**Figure 2.**
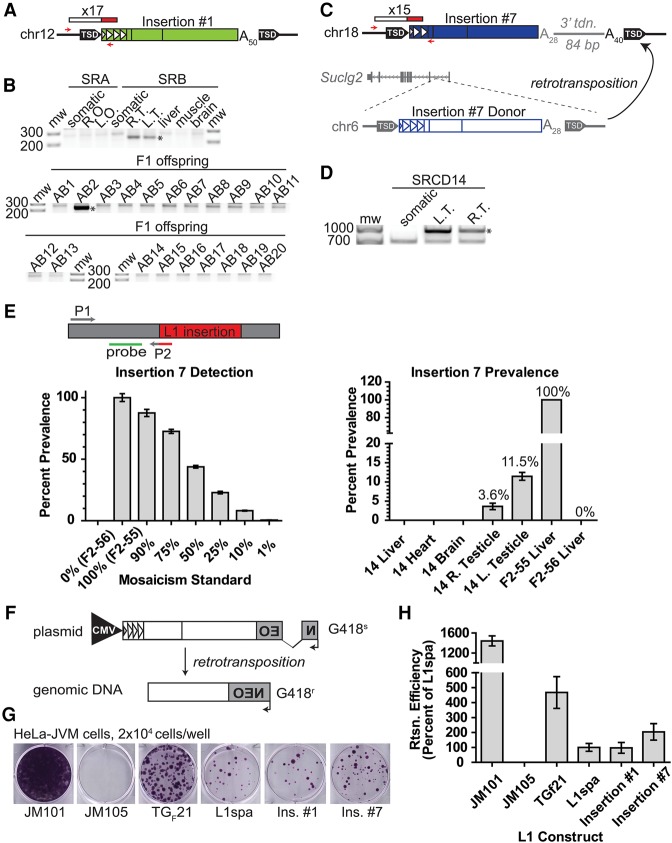
Retrotransposition and the generation of new active L1 copies in primordial germ cells. (*A*) Schematic of insertion #1. Red/white rectangle indicates mRC-seq reads. Red arrows indicate PCR primers used for genotyping. Triangles within the L1 5′ UTR represent T_F_ monomer units. (A_n_) poly(A) tract, (TSD) target-site duplication. (*B*) Genotyping panel for insertion #1. SRA (maternal) and SRB (paternal) tissues are indicated. (Somatic) Mix of liver, skeletal muscle, and brain genomic DNA; (R.O., L.O.) right ovary, left ovary; (R.T. and L.T.) right testicle, left testicle. F1 offspring of SRA and SRB (SRAB 1–20) were analyzed. Genotyping PCR was performed on liver gDNA for SRAB 1–9 and on whole embryo gDNA for SRAB 10–20. Here and in all subsequent figures, the validation product is marked with an asterisk. (*C*) Schematic of insertion #7 and its donor element on Chr 6. Features are annotated as in *A*; an 84-bp 3′ transduction of gDNA downstream of the Chr 6 donor element is represented in gray. The position of the donor element within the first intron of the gene *Suclg2* on Chr 6 is shown. (*D*) 5′ junction PCR validation for insertion #7. (*E*) A quantitative PCR (qPCR) assay for the prevalence of insertion #7. A forward primer (P1, gray) is situated within flanking genomic DNA, and a reverse primer (P2, gray/red) spans the junction between the 5′ end of the L1 and the genomic sequence. A hydrolysis probe (green) is situated within genomic sequence adjacent to the reverse primer and on the same strand. *Bottom*, *left*: the qPCR assay can detect mosaicism for insertion #7. The *x*-axis indicates the mosaicism standard used. The *y*-axis and percentages *above* each bar show detected percent prevalence, with the heterozygous animal set to 100%. Data are reported as the mean and standard deviation of four technical replicates per reaction. At *right*, the qPCR assay applied to somatic tissues (*x*-axis) and the germ cell fraction from the right and left testicles of SRCD14. Data are reported as the mean and standard deviation of three independent qPCR experiments, each comprising four technical replicates per reaction. (*F*) Rationale of the cultured cell retrotransposition assay (Moran et al. 1996; Wei et al. 2000). (*G*) Retrotransposition assay in human HeLa-JVM cells for insertion #1 and insertion #7. JM101 is a retrotransposition-competent human L1 (L1.3); JM105 is a negative control, consisting of L1.3 with a reverse transcriptase active site mutation. TG_F_21 is an active G_F_ subfamily mouse L1; L1_spa_ is an active T_F_ subfamily mouse L1. Colony formation indicates a successful retrotransposition event. (*H*) Quantification of the retrotransposition assay. L1_spa_ retrotransposition efficiency is set to 100%. Data are reported as the mean and standard deviation of three independent experiments (biological replicates), each of which comprised three technical replicates.

Next, we used PCR genotyping to investigate the developmental origin of each de novo L1 insertion (Supplemental Fig. S1B). Insertions #1 and #2 were identified by mRC-seq in mice SRAB2 and SRAB15, respectively (Fig. 2A; Supplemental Fig. S5A; Supplemental Table 2). We did not detect these insertions by PCR genotyping in the somatic tissues of parental mice SRA and SRB; however, insertions #1 and #2 were detected by PCR in both testicles of paternal mouse SRB (Fig. 2A; Supplemental Fig. S5A). Inheritance of each insertion by only 1/20 offspring, coupled with their presence in both testicles of the paternal mouse, suggested germline-restricted mosaicism for insertions #1 and #2 in mouse SRB (Fig. 2B; Supplemental Fig. S5A).

In an inverse approach to identify germline-restricted mosaic insertions, we performed deep (∼260×) mRC-seq on the germ cell fraction of each testicle of mouse SRCD14. We detected and PCR-validated insertion #7 in both testicles and did not detect it in the somatic tissues of SRCD14 (Fig. 2C,D; Supplemental Table 2). An insertion-specific genomic DNA qPCR assay targeting the 5′ L1-genome junction of insertion #7 revealed its prevalence of ∼11% and ∼4% in the germ cell fraction of the left and right testicle of SRCD14, respectively (Fig. 2E), and subsequent PCR genotyping demonstrated transmission of the insertion to 2/65 progeny of SRCD14 (F2-7 and F2-55) (Fig. 1; Supplemental Fig. S5B). As evidenced by our failure to detect L1 insertions #1, #2, and #7 from mesoderm-, ectoderm-, and endoderm-derived tissues of the respective paternal mice, we reasoned that these insertions likely arose in early primordial germ cells (PGCs) during paternal embryonic development, prior to PGC colonization of the genital ridge and the formation of the testes. Thus, we conclude that L1 retrotransposition can occur in early PGCs, resulting in germline-restricted genetic mosaicism and heritable de novo L1 insertions.

To investigate the capacity of de novo L1 insertions for subsequent retrotransposition, we cloned insertions #1 and #7 and tested their activity in a cultured cell retrotransposition assay (Fig. 2F; Moran et al. 1996; Wei et al. 2000). In HeLa cells, insertion #1 and insertion #7 jumped to ∼97% and ∼205% efficiency, respectively, relative to L1_spa_, a previously identified disease causing T_F_ insertion (Fig. 2G,H; Kingsmore et al. 1994; Mulhardt et al. 1994; Naas et al. 1998). Thus, consistent with previous reports (Naas et al. 1998; Kimberland et al. 1999), de novo L1 insertions are not only heritable but also have the potential to serve as progenitor elements for subsequent retrotransposition events.

**Figure 3.**
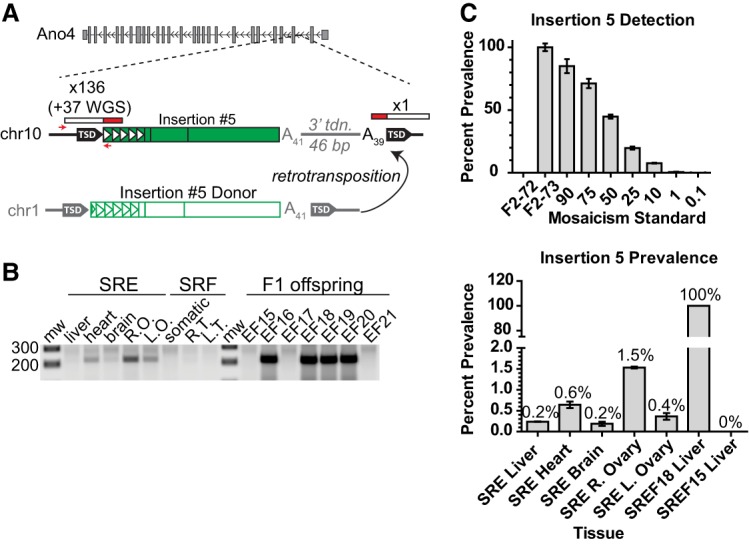
Retrotransposition in the early embryo. (*A*) Schematic of insertion #5 in the antisense orientation within the second intron of the gene *Ano4* on Chr 10, and the donor element of insertion #5 on Chr 1. Features are annotated as in Figure 2A. (*B*) Genotyping panel for insertion #5. SRE (maternal) and SRF (paternal) tissues are indicated. (Somatic) Mix of liver, heart, and brain genomic DNA; (R.O., L.O.) right ovary, left ovary; (R.T., L.T.) right testicle, left testicle. (*C*) *Top*: Control assay demonstrating the ability of the qPCR assay to detect mosaicism for insertion #5. Data are reported as the mean and standard deviation of four technical replicates per reaction. *Bottom*: Prevalence of insertion #5 among the tissues of maternal mouse SRE. Liver genomic DNA from mouse SREF18, a heterozygote for insertion #5, is set to 100%. Mouse SREF15, which lacks insertion #5, is included as a negative control. Data are reported as the mean and standard deviation of three independent qPCR experiments, each comprising four technical replicates per reaction.

We traced the developmental timing of six de novo L1 insertions to the early embryo. As an illustrative example, insertion #5 was detected by mRC-seq with a single sequencing read in the brain of maternal mouse SRE and robustly detected in 4/7 offspring (Fig. 1; Supplemental Table 2). PCR genotyping revealed bands of varying intensity among the somatic tissues and ovaries of mouse SRE (Fig. 3A,B), and by genomic DNA qPCR, the prevalence of insertion #5 ranged from ∼0.2% in brain to ∼1.5% in the right ovary (Fig. 3C). Similarly, insertions #3 (Supplemental Fig. S6A,B), #6 (Supplemental Fig. S6C,D), and #11 (Supplemental Fig. S6E) were each identified by mRC-seq in multiple offspring and genotyped as mosaic in the respective maternal tissues. Insertions #8 (Supplemental Fig. S6F) and #10 (Supplemental Fig. S6G) were identified by mRC-seq and were confirmed as mosaic in the tissues of mouse SREF17 and mouse F2-145, respectively, but were not transmitted to the limited offspring produced by these animals (five and six progeny, respectively) (Supplemental Fig. S6F,G). Therefore, we can neither confirm nor rule out the contribution of insertions #8 and #10 to the germ lineage. Taken together and consistent with previous studies (Garcia-Perez et al. 2007; van den Hurk et al. 2007; Kano et al. 2009; Wissing et al. 2012; Klawitter et al. 2016), our results demonstrate that L1 retrotransposition occurs in pluripotent cells of the early embryo, generates somatic and germline genetic mosaicism, and can give rise to heritable de novo L1 insertions.

**Figure 4.**
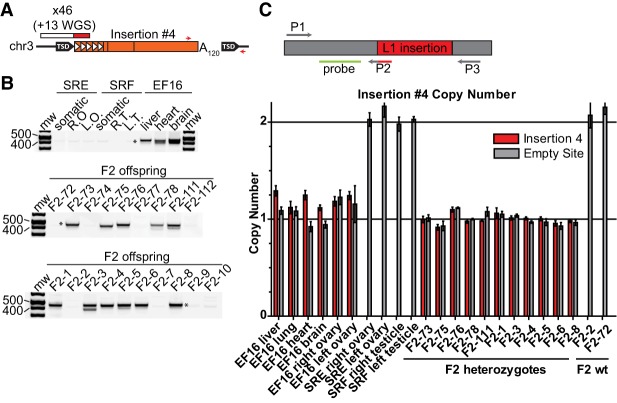
Retrotransposition in the late germline. (*A*) Diagram of insertion #4. Features are depicted as in Figure 2A. Red arrows indicate the position of 3′ junction genotyping primers. (*B*) Genotyping panel for insertion #4. SRE and SRF are P-generation mice, SREF16 is the F1 maternal mouse, and 72-78, 111-112, and 1-10 are the F1 offspring of SREF16 and SRCD14. (Somatic) Mix of liver, heart, and brain genomic DNA; (R.O., L.O.) right ovary, left ovary; (R.T., L.T.) right testicle, left testicle. Variation in size of the 3′ junction genotyping band likely reflects shortening of the poly(A) tract of insertion #4, as previously reported (Grandi et al. 2013). (*C*) Prevalence of insertion #4 among tissues of maternal mouse SREF16. *X*-axis, from *left*: tissues of F1 mouse SREF16, ovaries and testicles of P mice SRE and SRF, liver of 11 F1 mice heterozygous for insertion #4, and liver of two F1 mice lacking insertion #4. The average value among the 11 heterozygous F1 mice is set as a copy number of 1. Data are reported as the mean and standard deviation of three independent qPCR experiments, each comprising four technical replicates per reaction. (*Top*) Schematic of the probe-based qPCR assay used to quantify insertion #4 and empty site prevalence. The L1 insertion is shown in red; positions of the forward primer (P1) junction-spanning filled site reverse primer (P2), empty site reverse primer (P3), and hydrolysis probe (green) are indicated.

Of the remaining two de novo L1 insertions, insertion #4 likely represented a late germline event. This insertion was identified by mRC-seq in mouse SREF16, one of seven offspring of parental mice SRE and SRF, but was not detected in somatic and germ tissues of SRE or SRF (Fig. 4A,B; Supplemental Table 2). Upon crossing mouse SREF16 to mouse SRCD14, insertion #4 was transmitted to 11/19 offspring (Figs. 1, 4B), consistent with mouse SREF16 being either consummately heterozygous for the insertion or mosaic with a high degree of germline prevalence. To distinguish these possibilities, we used a 5′ L1-genome junction qPCR assay for insertion #4, with heterozygous and wild-type F2 offspring as controls, and found that SREF16 contained ∼1 copy each of insertion #4 and the genomic empty site across all tissues tested (Fig. 4C,D). Insertion #4 could not be detected in the gonads of SRE or SRF, despite sensitivity of the qPCR assay to 0.1% prevalence (Supplemental Fig. S7A). We therefore conclude that mouse SREF16 was heterozygous for insertion #4 and that this insertion arose sufficiently late during germline development of parental mouse SRE or SRF to preclude its detection in bulk gonad tissues, or possibly post-conception in SREF16 at the mature zygote stage.

Finally, insertion #9 was detected in 11/23 offspring of SRCD11 and SREF20 (Fig. 1; Supplemental Fig. S7B,C; Supplemental Table 2). This rate of transmission suggested either parental heterozygosity or mosaicism with a high degree of germline prevalence. However, we could not detect insertion #9 by nested PCR in the gonads of SRCD11 or SREF20 (Supplemental Fig. S7H). The developmental origins of insertion #9 are therefore unclear. We speculate that this event was germline-restricted mosaic in SRCD11 or SREF20, but that the subset of germ cells carrying insertion #9 was depleted by the time the gonads of these animals were harvested, at age 39 and 40 wk (Lei and Spradling 2013).

We next sought to identify the progenitor L1 elements responsible for heritable de novo insertions. During transcription of an RC-L1 element, the native L1 polyadenylation signal is occasionally bypassed in favor of a downstream genomic polyadenylation signal, and upon retrotransposition the nascent L1 insertion incorporates a genomic sequence tag, or 3′ transduction, that identifies the progenitor L1 element (Holmes et al. 1994; Moran et al. 1996, 1999; Goodier et al. 2000; Pickeral et al. 2000; Macfarlane et al. 2013). Two early PGC insertions (#2 and #7) (Fig. 2C; Supplemental Fig. S8A,C), one early embryonic insertion (#5) (Supplemental Fig. S8B), and two presumably recent polymorphic L1 T_F_ insertions differentially present/absent among our animals (Poly_L1T_F__3 and Poly_ L1T_F__4) (Table 1; Supplemental Table 2; Supplemental Figs. S3, S8D,E) carried 3′ transductions, implicating five distinct full-length L1 T_F_ elements present in the C57BL/6J reference genome. Thus, we have identified progenitor elements responsible for de novo heritable L1 insertions.

Having observed transmission of germline-restricted and somatic/germline mosaic insertions in mouse, consistent with previous studies of human patients (Brouha et al. 2002; van den Hurk et al. 2007), we next investigated a previously reported mutagenic human L1 insertion for which the developmental timing had not been resolved. The JH-27 insertion, which occurred in exon 14 of the Factor VIII gene, was identified in 1988 as the causative mutation in a case of noninherited hemophilia and was the first such example exhibiting L1 mobility in modern humans (Supplemental Fig. S9A; Kazazian et al. 1988). We performed a 5′ junction nested PCR (55 total cycles) specific for the JH-27 insertion on blood genomic DNA from the afflicted patient and his mother, with paternal DNA serving as a negative control. While a robust PCR product was detected in the patient DNA, the JH-27 insertion could not be detected in the maternal sample (Supplemental Fig. S9B). Given our mouse data indicating each heritable early embryonic L1 insertion was detectable in tissues derived from all three germ layers (Fig. 3; Supplemental Fig. S6), we suggest that insertion JH-27 was very likely maternal germline-restricted.

## Discussion

We uncovered 11 de novo L1 insertions among 85 mouse genomes, providing an estimate of one new insertion per eight mice (11/85 = 0.13 or ∼1/8). This figure is consistent with but more conservative than previous estimations that a new L1 insertion may arise in every two to three mice (Kazazian and Moran 1998; Kazazian 2000) and is much higher than estimates of one new L1 insertion per 100 live births in humans (Hancks and Kazazian 2012). Indeed, the technical hurdles limiting detection of 3′ L1-genome junctions may have precluded identification of additional, 5′ truncated de novo L1 insertions in our pedigrees. Furthermore, while 85 genomes constitute the largest cohort of individual mice examined for de novo L1 insertions to date, future examination of more animals may allow fine-tuning of this rate estimate. The structure of our breeding pedigrees allowed us to observe the transmission of two new L1 insertions, #4 and #5, from heterozygous animals to their offspring (Fig. 1). We found that transmission of these insertions to 58% and 52% of offspring, respectively, was not significantly different from the expected transmission rate of 50% (χ^2^ test with one degree of freedom, two-tailed *P*-values of 0.49 and 0.67, respectively). Thus, as expected, within a single generation we observe no evidence for positive or negative selection on a de novo L1 insertion. However, future studies tracking the transmission of de novo L1 insertions through many generations may reveal evidence for selection on particular insertions, perhaps dependent on their genomic locations and functional consequences.

**Figure 5.**
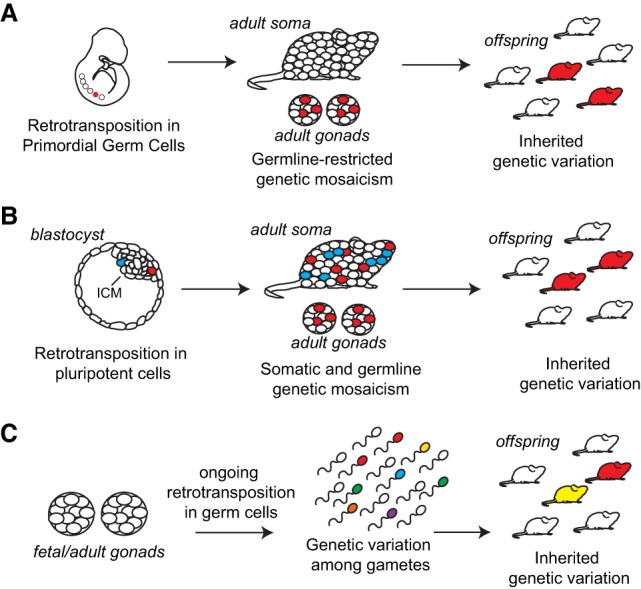
Model for the developmental origins of heritable L1 retrotransposition events. (*A*) Retrotransposition in the early primordial germline. From *left*: a retrotransposition event occurring in early PGCs (red) gives rise to germline-restricted genetic mosaicism in the adult animal. The L1 insertion is present in both testes and is heritable by subsequent generations, potentially by multiple F1 siblings. (*B*) Retrotransposition in the early embryo. Two retrotransposition events in pluripotent cells are indicated (red and blue). The red event contributes to somatic and germ tissues, while the blue event only contributes to somatic tissues in the adult animal. The red insertion is heritable by subsequent generations, potentially by multiple F1 siblings. (*C*) Retrotransposition in the late germline. Ongoing retrotransposition in adult germ cells, theoretically taking place at any stage from germline stem cells through mature gametes, may generate a large amount of diversity with individual insertions present at low frequency within the gamete pool (multicolored sperm). Each individual insertion has a low probability of contributing to genetic diversity in subsequent generations.

We established for the first time that heritable endogenous L1 retrotransposition events arise in early PGCs, before the PIWI/piRNA retrotransposon defense pathway becomes active in male embryonic gonads (Aravin et al. 2008), resulting in germline-restricted genetic mosaicism (Fig. 5A). Alternatively, it is possible that these insertions arose earlier during embryonic development, in cells of the primitive ectoderm that had been set aside for the germline and did not contribute to the somatic lineages (Soriano and Jaenisch 1986). It is worth noting that all three de novo insertions traced to early PGCs occurred in male mice. This correlation may stem from the relatively small number of insertions identified, and examination of more genomes may reveal equivalent events in female animals.

Consistent with previous studies (van den Hurk et al. 2007), we traced L1 insertions to the pluripotent cells of the early embryo and, in contrast to experiments using transgenic L1 reporter mice (Kano et al. 2009), we demonstrated germline transmission of four early embryonic insertions (Fig. 5B). All four transmitted insertions arose in somatic/germline mosaic female animals; again, future studies employing more animals may reveal transmission of similar events from mosaic males to offspring. Notably, deep mRC-seq of individual tissues from mosaic maternal mice SRA and SRE produced few reads (1–2) for insertions #3, #5, and #11 (Table 1; Supplemental Tables 1, 5), suggesting that additional mosaic insertions may have fallen below the detection threshold of mRC-seq. Thus, it is possible that early embryonic retrotransposition frequently generates low-level somatic-restricted mosaicism (Kano et al. 2009), as well as low-level somatic and germline mosaicism for insertions that ultimately fail to be transmitted. We therefore hypothesize that the early mammalian embryo is a complex mosaic incorporating a constellation of de novo retroelement insertions. We speculate that the contribution of a particular insertion to the germ lineage and ultimately its transmission to the next generation are collectively determined by the dynamics of cell fate specification in the early embryo, random chance, and perhaps whether the insertion has a functional impact on germ cell development and fertility. It is worth noting, for example, that somatic/germline mosaic insertion #5 exhibited a maximum prevalence of ∼1.5% in the right ovary of mosaic mouse SRE (Fig. 3C) but was transmitted to 4/7 (∼57%) of her offspring (Figs. 1, 3B; Supplemental Table 2). Thus, early embryonic insertions present at low prevalence in somatic and germ tissues of a parental mouse nevertheless have the potential to contribute substantially to genetic diversity in the next generation, presumably by their fortuitous presence in a subset of germ cells that ultimately give rise to offspring.

In contrast, insertions arising during later germline development (e.g., insertion #4) may contribute a broad spectrum of genetic diversity to the gamete pool, yet each insertion individually would have a small likelihood of transmission (Fig. 5C). Indeed, our experiments were primarily designed to identify transmitted de novo insertions in heterozygous offspring, and therefore our results may be biased toward early events (germline or embryonic) which ultimately are present in multiple gametes and therefore have a high likelihood of transmission. Thus, while an early embryonic or early PGC retrotransposition event may result in the same insertion being transmitted to multiple siblings, events occurring later in germline development may potentially produce more genetic diversity within a single generation.

De novo heritable L1 retrotransposition is a component of the ongoing evolutionary interplay between retroelements and mammalian genomes, the importance of which is exemplified by recent studies demonstrating the exaptation of LTR retrotransposon sequences and protein products for pluripotency maintenance and embryonic development (Wang et al. 2014; Grow et al. 2015). Furthermore, recent publications have implicated somatic retrotransposition in the brain as a feature of both normal neurobiology and neurological diseases (Muotri et al. 2005, 2010; Coufal et al. 2009, 2011; Baillie et al. 2011; Evrony et al. 2012, 2015; Bundo et al. 2014; Upton et al. 2015; Erwin et al. 2016). Early embryonic insertions contributing broad mosaicism to tissues including the brain likewise represent a component of genetic neurodiversity, and the prevalence and potential functional consequences of such insertions compared to those occurring specifically in cells of the neuronal lineage remain to be determined. Future studies employing single-cell genomic analyses, and other approaches, will likely further elucidate the scope and consequences of ongoing retrotransposition in the mammalian germline and early embryo (Malki et al. 2014).

## Methods

### Animals

All animal breeding and handling procedures were carried out in compliance with the guidelines set forth by the University of Queensland Animal Ethics Committee. To establish breeding pedigrees, adult wild-type C57BL/6J mice were ordered from the University of Queensland Biological Resources Facility (UQ-BRF), which in turn sources animals from the Animal Resources Center (ARC, Western Australia). The UQ-BRF and ARC provided helpful information regarding the source and breeding history of the animals used in this study. The “P-generation” mice used to initiate the breeding pedigrees in this study were no more than 10 generations removed from the Jackson Laboratory C57BL6/J strain.

### mRC-seq library construction

Genomic DNA from animals and tissues of interest was used to construct Illumina libraries for mRC-seq as described in Shukla et al. (2013), except using insert sizes of 450 and 550 bp. Illumina libraries were constructed using the Illumina TruSeq DNA LT kit or the Illumina TruSeq Nano DNA LT kit according to the manufacturer's instructions (Illumina). A detailed description of library preparation can be found in Supplemental Methods.

### mRC-seq hybridization reactions

Hybridization reactions were performed as described in Shukla et al. (2013), except using a pool of biotinylated capture probes designed against mouse retrotransposons represented by L1 subfamilies T_F_ G_F_ and A, SINEs B1 and B2, and the LTR elements IAP and ETn (Supplemental Table 1). Illumina libraries were pooled to achieve a total mass of 1 µg (Supplemental Table 1). A detailed description of mRC-seq hybridization can be found in Supplemental Methods.

### Illumina sequencing and mRC-seq analysis

We performed mRC-seq on either pooled genomic DNA from somatic tissues representative of the three embryonic germ layers (liver, skeletal or cardiac muscle, and brain) or DNA extracted from tail tips and ear punches (Supplemental Fig. S1B; Supplemental Table 1). We performed mRC-seq on the individual somatic tissues and gonads of mice SRA and SRE and the germ cell fraction of the left and right testicles of SRCD14. We also performed 30× whole-genome sequencing on P-generation animals SRE and SRF, and 22× WGS on their F1 offspring SREF15-SREF21, using pooled somatic tissue genomic DNA (Supplemental Table 1). Across all DNA sequencing libraries, we detected 92.8%, 97.8%, and 98.7% of recent reference genome L1 (T_F_, G_F_, A), LTR retrotransposon (IAP, ETn), and SINE (B1, B2) insertions, respectively (Supplemental Table 1).

A summary of library pooling and sequencing reads per library is included in Supplemental Table 1. mRC-seq libraries were sequenced on an Illumina HiSeq 2500 platform (Macrogen), generating 2×150-bp read pairs and 2×250-bp read pairs. Some libraries were also sequenced on an Illumina MiSeq platform, generating 2×250-bp and 2×300-bp read pairs. WGS libraries were sequenced on an Illumina HiSeq 2500 platform (Macrogen), generating 2×250-bp read pairs. mRC-seq and WGS data were processed as follows: read pairs were first trimmed from their 5′ and 3′ ends to remove any bases with quality <10, then assembled into contigs using FLASH (Magoc and Salzberg 2011) and default parameters. Two hundred fifty-mer reads from inserts >500 bp in length failed to form contigs but were retained and analyzed via the same process applied to contigs. Contigs were aligned to the mouse reference genome (mm10) using SOAP2 (Li et al. 2009) (parameters -M 4 -v 2 -r 1 -p 8). Only uniquely aligned reads were retained, and PCR duplicates were removed if they shared the genomic coordinates of another read. Unmapped reads were then aligned to a set of potentially active mouse retrotransposon consensus sequences obtained from Repbase (L1 T_F_, G_F_, and A subfamilies; SINE B1 and SINE B2; IAP LTR and ETn LTR) (Bao et al. 2015), using LAST (Kielbasa et al. 2011) (parameters –s 2 –l 12 –d 30 –q 3 –e 30). Reads aligned at >90% identity to a retrotransposon consensus sequence were retained if the alignment spanned ≥33 nt of one contig end. Reads passing this filter were then aligned to mm10 using LAST and formed into clusters following an existing strategy (Shukla et al. 2013). Clusters with ≥3 reads were then manually inspected for evidence of chimerism and annotated as polymorphic if found in an existing database of mouse polymorphisms (Nellaker et al. 2012) or in all of the libraries from at least one founder animal.

### Validation and structural characterization of de novo L1 insertions

Putative insertions were called as potentially de novo if they appeared in one or more offspring from the same pedigree and were absent from the parental mice. In addition, some insertions were readily detected in offspring and were also detected with 1–2 reads upon deep sequencing of individual tissues of maternal mice SRA and SRE. Such insertions were treated as potentially de novo and chimeric in the maternal mouse. Reads were then manually inspected using SerialCloner (http://serialbasics.free.fr/Serial_Cloner.html) and the BLAT tool on the UCSC Genome Browser (Kent 2002). Reads which clearly represented molecular chimeras and those which could not be manually assigned to a specific genomic location due to repeat content were disregarded. For putative insertions passing manual inspection, primers were designed in the putative 5′ and 3′ flanking genomic DNA (Supplemental Table 2). Oligonucleotide primers were ordered from Integrated DNA Technologies (IDT).

Empty-filled validation PCRs were carried out using primers specific to the 5′ and 3′ genomic sequence flanking putative insertions. Validation PCRs for 5′ and 3′ junctions were carried out using the appropriate flanking genomic primer paired with a primer internal to the L1 sequence; where necessary, hemi-nested and fully nested PCR reactions were carried out using appropriately designed genomic and L1-specific primers (Supplemental Table 2). The full details of PCR validation can be found in Supplemental Methods.

### Plasmid constructs

pTN201 (Naas et al. 1998), TG_F_21 (Goodier et al. 2001), pJM101/L1.3 (Dombroski et al. 1993; Sassaman et al. 1997), and pJM105/L1.3 (Wei et al. 2000) were described previously. Descriptions of these constructs can be found in Supplemental Methods.

### Generation of mouse L1 reporter constructs

Insertion #1 and insertion #7 were PCR-amplified using the Roche Expand Long Template PCR system and cloned into retrotransposition indicator vectors using standard molecular biology techniques. Detailed descriptions of the cloning strategies can be found in Supplemental Methods.

### Cultured cell retrotransposition assay

HeLa-JVM cells were seeded at 2 × 10^4^ cells/well in 6-well plates and transfected using FuGENE HD Transfection Reagent (Promega) at a ratio of 3 µL to 1 µg plasmid DNA. G418 selection (400 µg/mL) was initiated at 72 h post-transfection and carried out for 10–12 d (Wei et al. 2000).

Assays for transfection efficiency were performed in parallel by cotransfection of pCAG-EGFP with L1 reporter plasmids. At 48 h post-transfection, cells were subjected to flow cytometry on a Cyan ADP Analyzer (Beckman-Coulter) at the Translational Research Institute Flow Cytometry Core. The percentage of EGFP positive cells for each L1 reporter construct was used to normalize the G418-resistant colony counts obtained in the retrotransposition assay (Wei et al. 2000; Kopera et al. 2016). Full details of the cultured cell retrotransposition assay can be found in Supplemental Methods.

### Mosaicism analysis qPCR

Quantitative PCR using genomic DNA as template was carried out using primers and dual-labeled PrimeTime qPCR probes (5′ 6-FAM-ZEN-3′ Iowa Black FQ) from IDT. Control reactions for DNA input were performed using a predesigned PrimeTime qPCR assay for the single-copy mouse gene RPP25 (Mm.PT.58.21641426.g), with a dual-labeled probe (5′ 6-FAM™-ZEN-3′ Iowa Black FQ). Reactions were run on a Roche LightCycler 480 II with the following cycling conditions: 95°C, 5 min, followed by 45 cycles of 95°C for 10 sec, and 57°C for 1 min, then melt curve (0.11°C per sec from 57°C to 95°C). *C*_t_ values were calculated on the LightCycler software using absolute quantification 2nd derivative max. Details of mosaicism analysis qPCRs can be found in Supplemental Methods.

## Data access

mRC-seq and WGS data from this study have been submitted to the European Nucleotide Archive (ENA; http://www.ebi.ac.uk/ena/) under project accession number PRJEB10299. Sanger trace files from this study have been submitted to the NCBI Trace Archive (http://www.ncbi.nlm.nih.gov/Traces/home/index.cgi) with TI numbers TI2344112704–TI234412736 and TI234412752.

## Competing interest statement

J.A.J. is employed by Roche Sequencing Solutions, Inc., and Roche Sequencing Solutions reagents were used in the study.

## Supplementary Material

Supplemental Material
