# Supplementary material for: Heritable L1 retrotransposition in the mouse primordial germline and early embryo
Source: Genome Res. 2017 Aug;27(8):1395–405. doi: 10.1101/gr.219022.116 (PMC5538555; doi:10.1101/gr.219022.116)
Supplement: Supplemental Material [file supp_27_8_1395__index.html]

Heritable L1 retrotransposition in the mouse primordial germline and early embryo — Supplemental Material 

# Heritable L1 retrotransposition in the mouse primordial germline and early embryo

## Supplemental Material

- Supplemental\_fig\_S1.pdf
- Supplemental\_fig\_S8.pdf
- Supplemental\_table\_2.xlsx
- Supplemental\_fig\_S6.pdf
- Supplemental\_Methods.docx
- Supplemental\_fig\_S4.pdf
- Supplemental\_Table\_5.pdf
- Supplemental\_fig\_S2.pdf
- Supplemental\_fig\_S9.pdf
- Supplemental\_Table\_3.pdf
- Supplemental\_fig\_S7.pdf
- Supplemental\_table\_1.xlsx
- Supplemental\_fig\_S5.pdf
- Supplemental\_fig\_S3.pdf
- Supplemental\_Table\_4.pdf
