# Supplementary material for: Heritable L1 retrotransposition in the mouse primordial germline and early embryo
Source: Genome Res. 2017 Aug;27(8):1395–405. doi: 10.1101/gr.219022.116 (PMC5538555; doi:10.1101/gr.219022.116)
Supplement: Supplemental Material [file supp_gr.219022.116_Supplemental_fig_S1.pdf]

Supplemental Figure 1

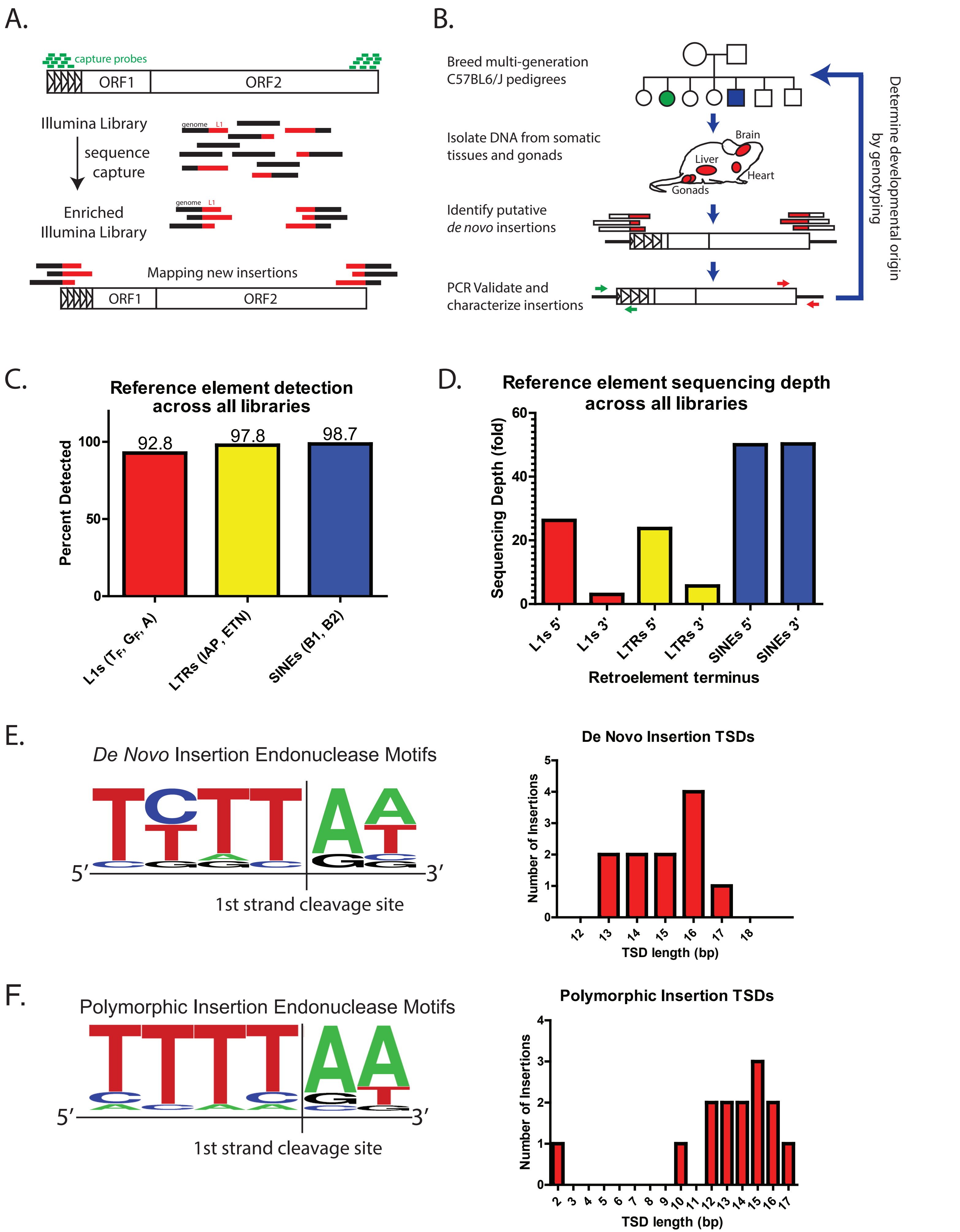

Supplemental Fig. 1 (cont'd)

G.

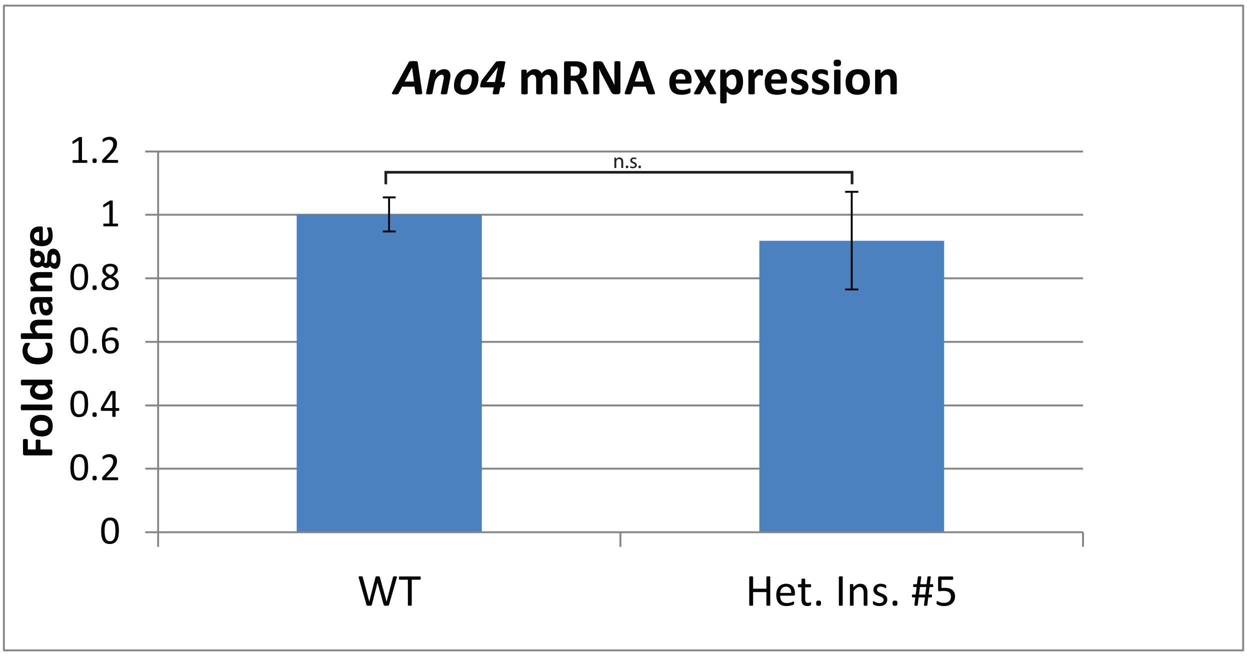

Supplemental Figure S1. Experimental approach and details of insertions.

A. Schematic of mRC-seq. Above: biotinylated capture probes (green rectangles) are tiled at the 5' and 3' termini of mouse mobile elements. Below: Illumina libraries are prepared from genomic DNA. Capture probes are used to enrich Illumina libraries for fragments containing the junctions between mobile elements and genomic DNA, enriched libraries are sequenced, and the mRC-seq bioinformatic pipeline is used to map putative insertions.

B. Experimental strategy: Two- and three-generation pedigrees of C57BL/6 mice were bred, and mRC-seq performed on genomic DNA isolated from somatic tissues (a pool of brain, liver, and heart or skeletal muscle genomic DNA). Putative insertions were identified bioinformatically and PCR validation was performed using primers spanning 5' and 3' L1-genome junctions, and in some cases pairs of flanking genomic primers were used to amplify the entire L1 insertion. 5' or 3' junction PCR was then used to genotype the insertion in the somatic and germline tissues of the mouse in which it was discovered and related mice, to determine the developmental origin of the insertion.

C. Reference retroelement detection across all libraries. Percentage of reference elements detected is shown on the y-axis. Retroelements are grouped as L1s (T_F, G_F and A subfamilies), LTRs (IAP and MusD), and SINEs (B1 and B2).

D. Sequencing depth of reference retroelements across all libraries. Fold sequencing depth is shown on the y-axis. The 5' and 3' junctions for reference L1s (T_F, G_F and A subfamilies), LTRs (IAP and MusD), and SINEs (B1 and B2) are indicated.

E. Endonuclease motif and TSD size distribution of *de novo* L1 insertions. The endonuclease motif graphic was generated using WebLogo (Crooks et al. 2004).

F. Endonuclease motif and TSD size distribution of polymorphic non-LTR retrotransposon insertions. This figure includes information from 14 polymorphic insertions for which complete structural hallmarks were elucidated using mRC-seq reads, PCR and capillary sequencing, or both. The endonuclease motif graphic was generated using WebLogo (Crooks et al. 2004).

G. Expression of *Ano4* mRNA in forebrain from mice lacking (WT) and heterozygous for (Het. Ins. #5) insertion #5. N=3 animals for both groups; heterozygote group comprised animals F2-149, F2-151, F2-152, and WT group comprised animals F2-147, F2-148, F2-150. p-value = 0.49, one-tailed t-test, indicating no significant difference in *Ano4* expression between mice heterozygous for and lacking insertion #5.
