# Supplementary material for: Heritable L1 retrotransposition in the mouse primordial germline and early embryo
Source: Genome Res. 2017 Aug;27(8):1395–405. doi: 10.1101/gr.219022.116 (PMC5538555; doi:10.1101/gr.219022.116)
Supplement: Supplemental Material [file supp_gr.219022.116_Supplemental_fig_S8.pdf]

Supplementary Figure 8A

Insertion #2 donor element: chr19:28149495-28156131

Black = Chr19 flanking sequence
Red = donor TSDs
Yellow = L1 sequence
Blue = 3' transduced sequence
Grey = original poly-A

5' junction
TTCAAAGAGGTTTAAAAAGTGTATTTAACTCTCTAATTCTCATCAAAGCTAAAGGTGTTTTTTTCATCTGAAGTAGC
CCTCCTC**TAAAATGTATTGTGA**Gttggtccgagacccgccgaacttaggaaattagtctgaacaggtgagagggtgc
gccagagaacctgacagcttctggaa

3'junction
aatgagctaaatacctaataaaaaaaaaatgaaaaaaaaaaaaaaaaaaaaaaaaa**taaaatGTATTGTGA**AATATT
ATGGAATGAATTATCTTGTGTATCTTTGAGGAACAAGATAATAAGATATATGTTTTTAATGCTG

Insertion #2

Black = Chr2 flanking sequence
Purple = Insertion #2 TSDs
Yellow = L1 sequence
Blue = 3' transduction
Grey = original poly-A
Green = new poly-A

5'junction
ACCTCTGATGTTTCTCTCATCAGCATAGAAGCATTTATGCTAAAGTCCATACCTAATAGGAGGAGCGGGAGCAGGCC
TTGATGTAGTTTGAAATTCTATT**AAAAATTTTTTTCTCT**cggctggggaggcgacctaagccacagcagcagcggtc
gccatcttggtccgggacccgccgaa

3'junction
gtgggtaaggggacttttggtatagcattggaaatgtaaatgagctaaatacctaataaaaaaaaaaatgaaaaaaa
aaaaaaaaaaaaaaaaaaaaaTAAAATGTATTGTGAAATAAAAAAAAAAAAAAAAAAAAAAAAAAAAAAAAAAAAAA
AAAAAAAAAAAAAAAAAAAAAAAAAAAAAACAAAAAAAAAAAAAAAAAAAAAAAAAAAAAAAAAAAAAAAAAAAAAAA
AAAAAAAAAAAAA**AAAAATTTTTTTCTCT**ATTCTTCACTATATTTCATTGAGCAATGGGCAGGAATGCATGCATAT
GTGTGTC

Supplementary Figure 8B

Insertion #5 donor element: chr1:7328682-7335740

Black = Chr1 flanking sequence
Red = donor TSDs
Yellow = L1 sequence
Blue = transduced sequence
Grey = original poly-A

5'junction
TAGTAGACACATGAGTAAATGAATAAAATAAATAATTCTCCAAATCTTAGAATCAAGATTGGCCATATCAGTCCAAT
ATTTATCAGGACATAACTATTCAACTTCTAAGGATT**AGAACTTTTTATCAG**atcttggtccgggacccgccgaactt
aggaaaTtagtctgaacaggtgagag

3'junction
ttggtatagcattggaaatgtaaatgagctaaatacctaataaaaaatggaaaaaaaaaaaaaaaaaaaaaaaaaaa
a**AGAACTTTTTATCAG**GTCAAATAAAGTGCAAGATGGGTTGCATCAATATTCCTGTTTTGCCAATAAGTCTGTCAGA
TATTCTGAGCCAGAAGTCCAAAGATGGATACTTCAACATTGCAAAGGAACCTTGAGTGATTATCTATGTATCTAGCA
ATCTCTGTCATGTCTTTGATTAGGATGG

Insertion #5:

Black = Chr10 flanking sequence
Purple = Insertion #5 TSDs
Yellow = L1 sequence
Blue = 3' transduction
Grey = original poly-A
Green = new poly-A

5'junction
TGCCACACAGATTTTGTACTATAGGAAAACAAAGAAAAAATACTTCAAGATATAAGCATATGGCAGATTTTTGTAAG
ATATCTTAGTAGAC**AAGAAAATAACAGC**ggggacagccggccaccttccggaccagaggacaggtgcccacccggct
ggggaggcggcctaagccacagcagcagcggtcgccatcttggtccgggatccgccgaactt

3'junction
ggccaaaggggggagtgggtgggtaggggagtgggggtgggtgggtaaggggacttttggtatagcattggaaat
gtaaatgagctaaatacctaataaaaaatggaaaaaaaaaaaaaaaaaaaaaaaaaaaaaaaaaaaaaaaaaGAACT
TTTTTTCAGGTCAAATAAAGTGCAAGATGGGTTGCATCAATAAAAAAAAAAAAAAAAAAAAAAAAAAAAAAAAAAAA
AAA**AAGAAAATAACAGC**AATAATTTATAAATGGAATCGTATGGCATGAAAAAGTGCCAGGATGGGAAAGGCAGTCAT
TCAGAGTGAAACAGAA

Supplementary Figure 8C

<u>Insertion #7 donor element: Chr6:95658065-95664762</u>

Black = Chr6 flanking sequence
Red = Donor TSDs
Yellow = L1 sequence
Blue = transduced sequence
Grey = original poly-A

5’junction
GAATCTCTTCTTAATTTTAGTTTCTAGTGGTGCCTAGATAGGTGTTTTGAATGATGAGATCTCACTCACGTGCCAATACT
GAGCACTCTTACCTGAGAGTGCTCATTACCACACTACCATTCAGCCGCAGTCGGATATTATGTCAGCTTGCTTAAG**ATGC**
CAATACAAGgcccggctggggaggcggcctaagccacagcagcagcggtcgccatcttggtccgagacccgccgaactta
ggaaattagtctgaacaggtgagagggtgcgccagagaacctgacagcttctggaacaggcagaggcacagaggcgctga

3’junction
gggaacgccagggccaaaggggggagtgggtgggtaggggagtgggggtgggtgggtaaggggggtcttttggtatagca
ttggaaatgtaaatgagctaaatacctaataaaaaatggaaaaaaaaaaaaaaaaaaaaaaaaaaa**ATGCCAATACAAG**T
GGGGTTTTTTTTTCAAAATGCGCTATCAATTACTTTTTAATTTATGTCAAAAGAATATAAAGAACAAGATGACAACCCAG
TAAATTACTTTTGTATTTTATCCAGTAATATCAACAGCATATCCTACCAAAACTATAAATCTCATTTTCAATGAAAAAGG
AATACATTTGGTTATGTTTACATCCATGAGTCATAATGTAAAGGAACTTACAAGAGATGATTATCCATTAACACAAATTA

<u>Insertion #7:</u>

Black = chr18 flanking sequence
Purple = Insertion #7 TSDs
Yellow = L1 sequence
Blue = 3’transduction
Grey = original poly-A
Green = new poly-A

5’ junction
ATTTTTTTAGGTGCTTCTCAGCTATTCGGTATTGAAGATGCTCCAACATGTAATAAGGACACATGCTCCATTACGTTCAT
CACAGCCTTGTTT**ACAATAGCCAGAAGC**gatcttggtccgggacccgccgaacttaggaaattagtctgaacaggtgaga
gggtgcgccagagaacctgacagcctctggaacaggcagaagcacagaggggctgaggcagcaccctgtgtgggccgggg

3’junction
agtgggggtgggtgggtaagggggtttttggtatagcattggaaatgtaaatgagctaaatacctaataaaaaatggaa
aaaaaaaaaaaaaaaaaaaaaaaaaATGCCAATACAAGTGGGGTTTTTTTTTCAAAATGCGCTATCAATTACTTTTTAAT
TTATGCCAAAAGAATATAAAGAACGAGATGAAAAAATAAAAAAAAAAAAAAAAAAAAAAAAAAAAAAAAAAA**ACAATAGCCA**
GAAGCTGGAAAGAACCCAGTTGTTTCTCAAAAGAGGAATGGATACAGAAAATTTGGTACATTTACACAATGGAGTACTAC

Supplementary Figure 8D

Poly L1TF 3 donor element: chr16:65055909-65062518

Black = Chr16 flanking sequence
Red = Donor TSDs
Yellow = L1 sequence
Blue = transduced sequence
Grey = original poly-A

5' junction
gagtgtctgaagacagctacagtgtacttacatataataaacaaacaaacaaataaataaataaataaataaattttaaaaa
gaaatatattttttgaaaTCTCTAAACAACAACAAAAAAGATTTCAACTTCCAGCAGACCTT**ACAAATGCAACAGTTG**Gaggaaatt
agtctgaacaggtgagagggtgcgccagagaacctgacagcttctggaacaggcggaagcacagaggcgctgaggcagcaccctttt
gtgggccggggacagccggccaccttccggaccagaggacaggtgcccacccggctggggaggcggcctaagccacagcagcagcg
gtcgccat

3'junction
agggccaaagggggggagtgggtgggtaggggagtgggggtgggtgggtaaggggggacttttggtatagcattggaaatgtaaatg
agctaaatacctaataaaaaatggaaaaaaaaaaagaaaaaaaataaaaaaaaaaaaaaaacaaaaaaaaaa**acaaaTGCAACAG**
TTGTCAATGTTTATCCTTTCAGGACCGAGTTAGGCAATGTTCTTCCTATCATAAAAGACTAAATACCGTGTTTAAATTGAACATGA
ATT

Poly L1TF 3:

Black = chr6 flanking sequence
Purple = Poly_L1TF_3 TSDs
Yellow = L1 sequence
Blue = 3'transduction
Grey = original poly-A
Green = new poly-A

5'junction
AATACATGAGCCTTTGAGGGACATTTGTATTCAGAACACCTTTGAGCTTGGTGCTTCTGTAATCTTGAAGGATACTTGGCAGAAAG
AGATGGTTTATGAGTAATGTCATAAT**AAGATGACAGACTC**Gacagcagcagcggtcgccatcttggtccgggacccgccgaactta
ggaaattagtctgaacaggtgagagggtgcgccagagaacctga

3'junction
GGTACTGTGCATGCTCCGGCCGCCATGGCGGCCGCGGGAATTCGATTGACTTTGTGTGCCCCGGTACAGGGGAACGCCAGGGCCAA
AGGGGGGGAGTGGGTGGGTAGGGGAGTGGGGGTGGGTGGGTAAGGGGGACTTTTGGTATATCATTGGAAATGTAAATGAGCTAAAT
ACCTAATAAAAAATGGAAAAAAAAAAAGAAAAAAAAATAAAAAAAAAAAAAAAAAAACAAAAAAAAAAACAAATGCAACAGTTGTC
AATGTTTATCCTTTCAGGACCGAGTTAGGCAATGTAAAAAAAAAAAAAAAAAAAAAAAAAAAAAAAAAAAAAAAAAAAAAAAAAAA
AAAAAAAAAAAAAAAAAAAAAAAAAAAAAAAAA**AAGATGTCAGACTC**TGTTCTTACTGTGTGTGTGTTGGTTGAGTGTGACATGAC
AAGTGGTGACTGAGTCACAGTGAGCCTGG

Supplementary Figure 8E

PolyL1TF_4 Donor element: Chr7:58421728-58428359

Black = Chr7 flanking sequence
Red = Donor TSDs
Yellow = L1 sequence
Blue = transduced sequence
Grey = original poly-A

5’junction
CAAGCCAACATAAAAGCAGATCTTTCAGCATGATTTACAAGGACAGAGATTTATTGTCATAGCTCAGGCAGAAGTAC
AAGCTGTACTTAAAAATATCTGGAACTTTGTGTAAAAT**AAAAAATGTC**Gaggtgagagggtgcgccagagaacctga
cagtctctggaacaggcagaagcacagaggggctgaggcagcaccc

3’junction
ggggactttttggtatagcattggaaatgtaaatgagctaaatacctaataaaaaatggaaaaataaaaaataaaat
aaaataaaaaaaaaaaaaaaagaaa**aaaaaaTGTC**TGAAATGAACCTTTAAGGATAATGTACAGAATATAAAGAAAA
TAAGGGCACCCCCTCATGGACAGAGCTGCAAGGCACAAGGCATAGGGATTTTTAGGACAAAATAGCAGGTGTTACCA
AAACACACAAAATGGAGAGGGTGGCTGAAGATGTGACCAATAAAAGAAACTGGGAAATGGATGAATCAT

Poly L1TF 4:

Black = chr17 flanking sequence
Purple = Poly_L1TF_4 TSDs
Yellow = L1 sequence
Blue = 3’transduction
Grey = original poly-A
Green = new poly-A

5’ junction
CAAACTATATTTGCAGTCAAATTTTAAATTAAATGGGGATATAGTT**GAAAAAGAAAATACAG**ATCTTGGTCCGGGAC
CCGCCGAACTTAGGAAATTAGTCTGAACAGGTGAGAGGGTGCGCCAGAGAACCTGACAGCTTCTGGAACAGGCGGAA
GCACAGAGGCGCTGAGGCAGCACCCTTTGTGGGTCGGGGACAGCCAGCCACCGTCCGGACCGGAGGACAGGTGCCCG
CCCGGCTGGGGAGGCGGCCTAAGCCAC

3’junction
GGGGGGGAGTGGGTGGGTAGGGGAGTGGGGGTGGGTGGGTAAGGGGGACTTTTGGTATATCATTGGAAATGTAAATG
AGCTAAATACCTAATAAAAAATGGAAAAATAAAAAATAAAATAAAATAAAAAAAAAAAAAAAAAGAAAAAAAAATGTC
TGAAATGAACCTTTAAGGATAATGTACAGAATATAAAGAAAATAAAGGCACCCCCTCATGCACATATCTGCAAGGCA
CAAAGCATACGGATTTTTAAGACAAAATATCATGTGTTACCAAAACACACAAAATAAAAAAAAAAAAAAAAAAAAAA
AAAAAAAAAAAAAAAAAAAAAAAAAAAAAAAAAAAAAAAAAAAAAAAAAAAAAAAAAAAAAAAAAAAA**GAAAAGAA**
AATACAGCAAGTTGGTAATCTATAAATGAAATAGTGTAATATAAGCAAAATGCTTTATATAATAAGGAACAGGTTAA
ACATTAGGGAATATTGGTATTGTATAGAGGAATAGCAGTAGTACTGAATCAGAAAATAACACTGGGGTTAA

Supplemental Figure S8. Analysis of 3' transduction-bearing insertions

A. Characterization of the 3' transduction carried by insertion #2 and identification of the donor element. Above, the 5' and 3' termini of the donor element are shown. Genomic DNA is shown in black text, TSDs belonging to the donor element in red, L1 sequence in yellow, the donor poly(A) tract in green and the transduced sequence in blue. A putative poly(A)denylation signal is underlined. Below, the 5' and 3' termini of insertion #2 are shown. Genomic DNA is shown in black text, TSDs flanking insertion #2 in purple, L1 sequence in yellow, the donor poly(A) tract in green, the 3' transduction in blue, and the poly(A) tract of insertion #2 in grey. Poly(A) tract lengths were determined by capillary sequencing and should be regarded as estimates.

B. Characterization of the 3' transduction carried by insertion #5 and identification of the donor element.

C. Characterization of the 3' transduction carried by insertion #7 and identification of the donor element.

D. Characterization of the 3' transduction carried by Poly_L1TF_3 and identification of the donor element.

E. Characterization of the 3' transduction carried by Poly_L1TF_4 and identification of the donor element.
