# Supplementary material for: Heritable L1 retrotransposition in the mouse primordial germline and early embryo
Source: Genome Res. 2017 Aug;27(8):1395–405. doi: 10.1101/gr.219022.116 (PMC5538555; doi:10.1101/gr.219022.116)
Supplement: Supplemental Material [file supp_gr.219022.116_Supplemental_fig_S6.pdf]

Supplemental Figure 6

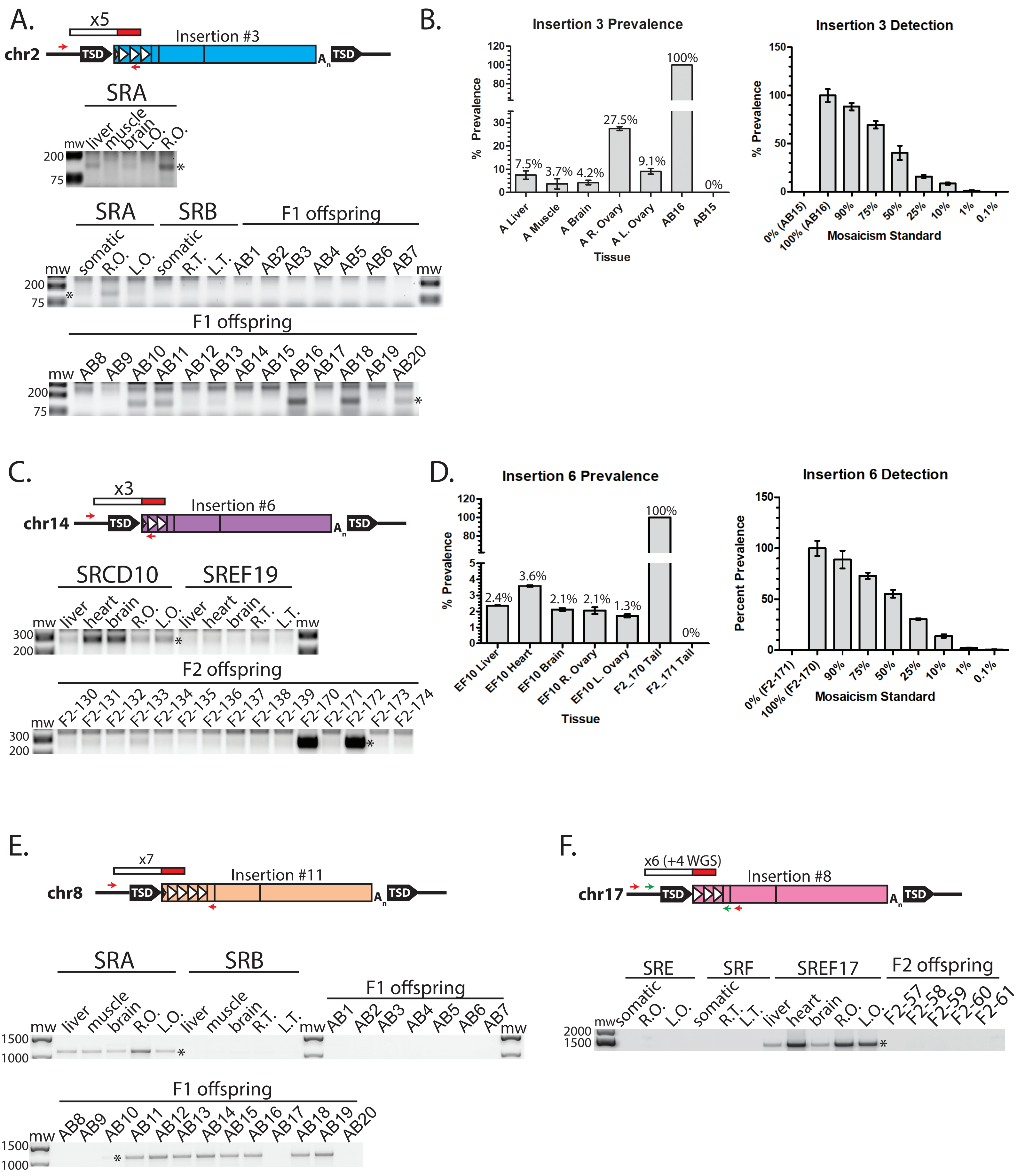

Supplemental Figure 6 (cont'd)

G.

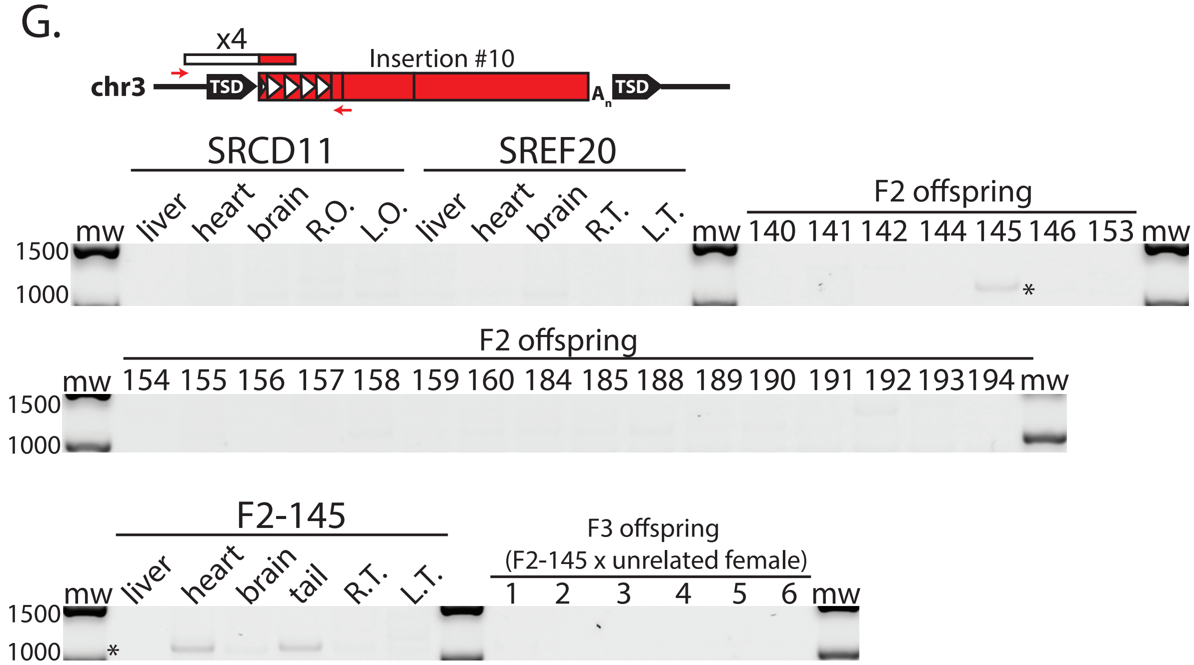

Supplemental Figure S6. Insertions arising in the early embryo.

A. Above: schematic of insertion #3, annotated as in Fig. 2A. Red arrows indicate the position of validation PCR primers used to genotype the insertion. Below: genotyping panel. The first gel panel shown the individual somatic tissues and ovaries of SRA. The second gel panel shows the mixed somatic tissues (liver, brain, skeletal muscle) of SRA and SRB, the gonads of SRA and SRB (R.O. and L.O. = right ovary and left ovary; R.T. and L.T. = right testicle and left testicle) and the F1 offspring of SRA and SRB. As in Fig. S5A, liver gDNA was used to genotype SRAB1-SRAB9, and whole embryo gDNA was used for SRAB11-SRAB20.

B. Quantitative PCR on genomic DNA for the percent prevalence of insertion #3. Right: Control qPCR assay to demonstrate the ability of the technique to quantify mosaicism for insertion #3. The y-axis shows percent prevalence of the insertion in each reaction, setting heterozygous mouse SRAB16 to 100%. Data are reported as the mean and standard deviation of four technical replicates per reaction. Left: qPCR assay for mosaicism of mouse SRA. Percent prevalence for insertion #3 is shown on the y-axis; somatic tissues and ovaries of mouse SRA are shown on the x-axis. Liver gDNA from heterozygous mouse SRAB16 was set to 100% prevalence. Data are reported as the mean and standard deviation of three independent qPCR experiments, each comprising four technical replicates per reaction.

C. Above: Schematic of insertion #6, annotated as in Fig. 2A. Red arrows indicate the position of 5' junction genotyping primers. Below: genotyping panel for insertion #6 in the somatic tissues and gonads of F1 mice SRCD10 and SRCD19, and in tail gDNA from F2 mice 130-139 and 170-174.

D. Quantitative PCR on genomic DNA for the percent prevalence of insertion #6. Control assay (right) and assay to determine the prevalence of insertion #6 among the somatic tissues and gonads of SREF10 were carried out as in Extended Data Fig. 6B. Percent prevalence was calculated relative to heterozygous mouse F2-172.

E. Above: Schematic of insertion #11, annotated as in Fig. 2A. Red arrows indicate the position of 5' junction PCR primers used for genotyping. Below: genotyping panel for insertion #11 among the somatic tissues and gonads of SRA and SRB, and among F1 offspring SRAB1-SRAB20.

F. Above: Schematic of insertion #8, annotated as in Fig. 2A. The position of nested 5' junction genotyping PCR primers is indicated (red arrows = outer primers, green arrows = inner primers). Below: genotyping panel for insertion #8 on somatic tissues (mixture of liver, brain, and heart gDNA) and gonads (R.O. and L.O. = right ovary and left ovary; R.T. and L.T. = right testicle and left testicle) of mice SRE and SRF, the somatic tissues and ovaries of F1 mouse SREF17, and the F2 offspring of SREF17 (57-61).

G. Above: schematic of insertion #10, annotated as in Fig. 2A. PCR genotyping primers are indicated as red arrows. Below: genotyping panel for insertion #10 among the somatic tissues and gonads of SRCD11 and SREF20, and among tail gDNA from the F2 offspring of SRCD11 and SREF20. The second gel panel shows genotyping of the somatic tissues and gonads of mouse F2-145, and genotyping of liver gDNA from six F3 offspring of F2-145. F3 offspring were generated by mating F2-145 with an unrelated female mouse.
