# Supplementary material for: Heritable L1 retrotransposition in the mouse primordial germline and early embryo
Source: Genome Res. 2017 Aug;27(8):1395–405. doi: 10.1101/gr.219022.116 (PMC5538555; doi:10.1101/gr.219022.116)
Supplement: Supplemental Material [file supp_gr.219022.116_Supplemental_Methods.docx]

**Supplemental Methods**

**Tissue Collection and Genomic DNA Extraction:** Genomic DNA was collected from live mice by tail tipping or blood collection. Mice were euthanized by cervical dislocation or carbon dioxide, dissected, and liver, heart, brain, skeletal muscle, lung, tail, ovaries, and testicles were collected. The germ-cell fraction was prepared from the testicles of mouse CD14 as described by Chang *et al.*(Chang et al. 2011). Genomic DNA was extracted from tissues of interest by either standard phenol-chloroform extraction, by using a Qiagen Blood and Tissue DNA Midi Kit following manufacturer’s instructions, or by using a QIAamp DNA Mini Kit following manufacturer’s instructions (Qiagen Hilden, Germany). DNA was eluted in molecular grade water and concentration measured by Qubit dsDNA high-sensitivity assay kit on a Qubit HS DNA Fluorometer (Life Technologies Carlsbad, CA).

**mRC-seq Library Construction:** genomic DNA was sheared using a Covaris M220 Focused Ultrasonicator in 130 μl microTUBE AFA fiber snap-cap vials (Covaris Woburn, MA). For 450 bp insert libraries, the following ultrasonicator parameters were used: 50 Watts, duty factor 10%, 200 cycles per burst, duration 70 seconds. For 550 bp insert libraries, the following parameters were used: 50 Watts, duty factor 10%, 200 cycles per burst, duration 50 seconds. DNA was concentrated and fragments <100 bp were removed using Agencourt AMPure XP beads (Beckman Coulter, Brea, CA) according to manufacturer’s instructions, using a 1:1.1 ratio of DNA to beads. Sheared DNA concentration was measured by Qubit dsDNA high-sensitivity assay kit and concentration and fragment size were confirmed on an Agilent 2100 Bioanalyzer using an Agilent DNA 1000 chip and reagents according to manufacturer’s instructions (Agilent Technologies, Santa Clara, CA).

Illumina libraries were constructed using the Illumina TruSeq DNA LT kit or the Illumina TruSeq Nano DNA LT kit according to manufacturer’s instructions (Illumina San Diego, CA), using indexed adapters to allow multiplexing. Libraries were run on a 2% high-resolution agarose gel (Sigma, St. Louis, MO) pre-stained with SYBR Gold Nucleic Acid Gel Stain (Life Technologies). Gels were visualized and gel fragments excised on Safe Imager 2.0 Blue Light Transilluminator (Life Technologies). For 450 bp insert libraries, the target gel fragment size was 550-570 bp. For 550 bp insert libraries, the target gel fragment size was 650-670 bp. DNA was purified from gel slices using a MinElute Gel Extraction Kit (Qiagen) according to manufacturer’s instructions.

Purified library fragments were subjected to ligation mediated PCR (LMPCR). Reactions consisted of 50 μl Phusion High-Fidelity Master Mix with HF Buffer (New England Biolabs Ipswich, MA), 1 μl of 100 μM TruSeq Forward Primer (TS-F) 5′-AATGATACGGCGACCACCGAGA, 1 μl of 100 μM TruSeq Reverse Primer (TS-R) 5′-CAAGCAGAAGACGGCATACGAG, and molecular grade water to 100 μl total volume. Cycling conditions were as follows: 98°C for 45 sec, followed by 8 cycles of: 98°C for 10 sec, 60°C for 30 sec, and 72°C for 30 sec, followed by 72°C for 5 min and 12°C hold. Samples were cleaned with AMpure XP beads (Beckman Coulter) following manufacturer’s instructions, using a 1:1.1 ratio of DNA to beads. Samples were eluted in molecular grade water and quantified using the Bioanalyzer DNA 1000 chip (Agilent Technologies) according to manufacturer’s instructions.

**mRC-seq Hybridization Reactions:** Illumina libraries were pooled to achieve a total mass of 1 μg. A total of 10 μl of 100 μM index-specific 3'-dideoxy cytosine terminated (3'ddC) blocking oligos, 10 μl of Universal Blocking Oligo (AATGATACGGCGACCACCGAGATCTACACTCTTTCCCTACACGACGCTCTTCCGATC/3'ddC/) (SeqCap EZ HE-Oligo Kits A and B, Roche NimbleGen Madison, WI), and 10 μl of SeqCap Easy Developer Reagent (Roche NimbleGen) were added to each pool. Library/blocking oligo pools were dried down in a speed-vac (70°C, 30 min). To each dried-down sample, 7.5 μl Hybridization Buffer and 3 μl Component A (Roche NimbleGen) were added. Samples were spun down briefly and then heated to 95°C for 5 minutes, with a 95°C heated block placed on top of the sample tube to prevent evaporation. The entire sample was then transferred to a 0.2 ml PCR tube containing 4.5 μl of mRC-seq biotinylated probe pool. The sample-probe mix was placed in a thermocycler pre-heated to 95°C (lid temperature 105°C) for 3 minutes, then immediately transferred to a 47° thermocycler (lid temperature 57°C) and held for 3 days.

Hybridization reactions were washed according to the SeqCap SR User’s Guide (Roche NimbleGen), and eluted in 50 μl of molecular-grade water. Two post-hybridization LMPCR reactions were performed for each sample, consisting of 25 μl sample, 50 μl Phusion High-Fidelity Master Mix with HF Buffer (New England Biolabs), 1 μl of 100 μM TruSeq Forward Primer (TS-F), 1 μl of 100 μM TruSeq Reverse Primer (TS-R), and molecular grade water to 100 μl total volume. Cycling conditions were identical to pre-hybridization LMPCR as described above. The two PCR reactions for each sample were then pooled, and cleaned up using Agencourt AmPure XP Beads (Beckman Coulter) at a 1:1.1 ratio of DNA to beads and eluted in 15 μl of Elution Buffer (Qiagen). Samples were quantified and fragment size confirmed using a Bioanalyzer DNA 1000 chip (Agilent Technologies).

**Validation and Structural Characterization of *de novo* L1 Insertions:** Empty-filled validation PCRs were carried out using the Roche Expand Long Template PCR System dNTP Pack (Roche Cat# 04829069001). Each reaction consisted of 5 μl 5x Buffer with 12.5 mM MgCl_2_, 1.25 μl DMSO 100%, 1.25 μl 10 mM dNTPs, 1 μl primer mix (25 μM each primer, genomic 5' and genomic 3'), 1.75 units Expand Long Template enzyme mix, 2-50 ng genomic DNA template, and molecular grade water to a final volume of 25 μl. Cycling conditions were as follows: 92°C for 3 minutes, followed by 10 cycles of 92°C for 30 seconds, 60°C for 30 seconds, and 68°C for 7.5 minutes, then followed by 30 cycles of 92°C for 30 seconds, 58°C for 30 seconds, and 68°C for 7 minutes; increasing the 68°C extension step by 20 seconds each cycle, then followed by a 68°C final extension of 10 minutes and a final hold of 12°C.

The 5' and 3' L1-genome junction validation PCRs were carried out using MyTaq HS DNA polymerase (Bioline, London, UK) with the following reaction components: 5 μl 5x MyTaq reaction buffer (including dNTPs), 1 μl primer mix (25 μM each primer, genomic flank and corresponding L1-specific primer), 1 unit MyTaq enzyme, 2-50 ng of genomic DNA template and molecular grade water to 25 μl. Cycling conditions were as follow: 95°C for 1 min, then 35 cycles of 95°C for 30 sec, 57°C for 15 sec, 72°C for 10 sec, followed by final extension at 72°C for 10 min, then hold at 12°C. Nested and hemi-nested PCR reactions were carried out using cycling conditions described above, with the first-round (outer) PCR comprising 20 cycles, followed by ExoSAP-IT clean-up according to manufacturer’s instructions (Affymetrix). 2μl of cleaned-up PCR product was used in the second-round PCR, which consisted of 35 cycles.

PCR products were run on a 1% (empty-filled) or 2% (junction product) agarose gel (Bioline) stained with SYBR-safe Nucleic Acid Gel Stain (Life Technologies). Gels were imaged using a Typhoon FLA 9000 (GE Healthcare Life Sciences, Buckinghamshire, UK) and gel fragments excised on a Safe Imager 2.0 Blue Light Transilluminator (Life Technologies). DNA was extracted from gel slices using the QiaAmp Min-elute Kit (Qiagen) following manufacturer’s instructions. Filled-site PCR products were cloned using the TOPO-XL PCR cloning kit (Life Technologies) and junction products were cloned using the pGEM-T Easy system (Promega, Madison, WI). Cloned junction PCR fragments were Sanger sequenced to resolve insertion structure. Cloned filled-site PCR products in TOPO-XL vectors were Sanger sequenced with M13 forward and reverse primers to confirm the presence of retroelement sequence, and then 3 to 5 clones per element were subjected to NEB-Next Ultra DNA Library Kit (New England Biolabs) preparation, sequenced on an Illumina MiSeq, and reads were assembled to fully elucidate the sequence of each clone. From the 3-5 clones for each insertion, a consensus was constructed using BioEdit software (http://www.mbio.ncsu.edu/bioedit/bioedit.html), allowing discrimination of PCR-induced mutations.

**Plasmid Constructs:**

pTN201: was described previously (Naas et al. 1998). It consists of the pCEP4 backbone (Life Technologies) containing L1_spa_, a retrotransposition-competent mouse L1 of the L1MdTf subfamily and the *mneoI* retrotransposition indicator cassette (Freeman et al. 1994). The *mneoI* retrotransposition indicator cassette comprises the neomycin phosphotransferase gene (neo) in the antisense orientation to the upstream L1 element, driven by an SV40 promoter. The neo gene is interrupted by an intron in the same transcriptional orientation as the upstream L1 element. This arrangement ensures that transfected cells receive an intact, spliced copy of the neo gene only upon a successful round of retrotransposition of the L1 element (Moran et al. 1996).

TG_F_21: was described previously (Goodier et al. 2001). It consists of the pCEP4 backbone containing TG_F_21, a retrotransposition-competent L1 of the L1MdG_F_ subfamily and the *mneoI* retrotransposition indicator cassette.

pJM101/L1.3: was described previously (Dombroski et al. 1993; Sassaman et al. 1997). It consists of the pCEP4 backbone containing L1.3, a retrotransposition-competent human L1, and the *mneoI* retrotransposition indicator cassette.

pJM105/L1.3: was described previously (Wei et al. 2000). It contains the retrotransposition-competent L1.3 with a missense mutation in the reverse transcriptase domain of ORF2 (D702A) and the *mneoI* retrotransposition indicator cassette.

**Generation of mouse L1 Reporter Constructs:** all restriction enzymes used for cloning were obtained from NEB. Insertion #1 was amplified from genomic DNA of mouse SRAB2 using a forward primer spanning the junction between genomic sequence and the 5' end of the L1 sequence, and a genomic reverse primer, using the Roche Expand Long Template PCR system with the same cycling conditions described above for empty-filled validations. The forward primer introduced a NotI site at the 5' end of the PCR product. This PCR product and the L1_spa_ reporter construct pTN201 were digested with NotI and AleI, and the Insertion #1 PCR product was ligated into the pTN201 backbone. The resulting plasmid contained the sequence of Insertion #1 from the NotI site through AleI, but L1_spa_ sequence from the AleI site (near the end of orf2) through the 3'UTR. To replace the 3' end of this chimeric element (pCEP4_Ins1_spa3'UTR_*mneoI*) with Insertion #1 sequence, a forward primer containing the AleI site and a reverse primer annealing to the 3' end of the Insertion #1 3'UTR and introducing a PacI site were used to amplify the corresponding fragment of Insertion #1 from the TOPO-XL clone of Insertion #1 described above. This PCR fragment and pCEP4_Ins1_spa3'UTR_*mneoI* were digested with AleI and PacI, and the PCR fragment ligated into the pCEP4_Ins1_spa3'UTR_*mneoI* backbone, giving rise to pPG_Ins1_*mneoI*.

Insertion #7 was amplified from genomic DNA of mouse F2-55, using Roche Expand Long Template PCR system with cycling conditions as described above. A forward primer that introduced a NotI site was designed in the 5' flanking genomic DNA, and used in conjunction with a reverse primer spanning the junction between the 3' end of the insertion (poly-A tail) and flanking 3' genomic DNA. PCR products were subjected to NEB-next library preparation and thoroughly sequenced on a MiSeq as described above, and cloned into TOPO-XL. To generate the L1 reporter construct, a “custom 3'UTR” intermediate cloning vector was prepared. The 3'UTR region of each insertion was amplified from its TOPO-XL clone using a forward primer introducing a NotI restriction site and encompassing the existing SfiI restriction site in L1(5'-NotI-SfiI-[L1sequence]-3'), and a reverse primer near the end of the 3'UTR, consisting of 5'-PacI-BamHI-[L1 sequence]-3'. This product was digested with NotI and BamHI, and ligated into pCEP4. The resulting pCEP4-3'UTR clone was transformed into Dam-/Dcm- chemically competent *E. coli*, and non-methylated plasmid preps generated. A PCR product amplified from genomic DNA as described above was double-digested with NotI and the methylation-sensitive restriction endonuclease SfiI, and ligated into the NotI-SfiI digested non-methylated pCEP4-3'UTR backbone. The resulting construct was digested with NotI and PacI, in parallel with pCEP4-L1_spa_, and the new L1 insertion was ligated into the neomycin resistance retrotransposition indicator backbone, giving rise to pPG_ins7_*mneoI*.

As PCR amplification introduces random mutations, at least 6 PCR products for each insertion described above were cloned into the retrotransposition indicator backbone and tested in the cultured cell retrotransposition assay (below). Some of the constructs proved incompetent for retrotransposition so the best jumper for each insertion was considered to be representative of that element.

**Cultured cell retrotransposition assay:** HeLa-JVM cells were seeded at 2 x 10^4^ cells/well in 6-well plates in DMEM complete medium (10% FBS, 1X penicillin/streptomycin, 1X Glutamax, Life Technologies). Transfection was performed using 1 µg plasmid DNA and 3 µl FuGENE HD Transfection Reagent (Promega) in 97 µl of Opti-MEM (Life Technologies) per well. The FuGENE/Opti-MEM/DNA-mixture was added to each well containing 2 ml DMEM-complete medium and the plates were incubated at 37°C and 5% CO_2_. The culture medium was replaced 16 hours post-transfection. G418 selection (400 µg/ml) was initiated at 72 hours post-transfection. 10-12 days later, G418 positive foci were fixed in 2% formaldehyde, 0.2% glutaraldehyde (Sigma-Aldrich, St. Louis, MO, USA) and 1x PBS at RT for 30 min and then stained with 0.1 % crystal violet solution (Sigma-Aldrich).

Assays for transfection efficiency were performed in parallel. Per well, 0.5 μl of each L1 reporter plasmid was co-transfected with 0.5 μl pCAG-EGFP. Duplicate wells were transfected for each L1 reporter plasmid, and untransfected wells were maintained as a negative control. 48 hours post-transfection, cells were harvested by trypsinization and subjected to flow cytometry on a Cyan ADP Analyzer (Beckman-Coulter). The percentage of EGFP positive cells for each L1 reporter construct was used to normalize the G418-resistant colony counts obtained in the retrotransposition assay (Kopera et al. 2016).

**Mosaicism Analysis qPCR:** Assays were designed so that the forward primer and dual-labelled probe anneal to 5' flanking genomic DNA, and the reverse primer spans 5' the L1-genome junction. For each template, a control assay to account for differences in the amount of input DNA was performed in parallel using IDT pre-designed PrimeTime qPCR assay for the single-copy mouse gene RPP25 (Mm.PT.58.21641426.g), with a dual-labelled probe (5' 6-FAM™-ZEN-3' Iowa Black® FQ). Reactions were run on a Roche LightCycler 480 II with the following cycling conditions: 95°C, 5 min., followed by 45 cycles of 95°C for 10 sec. and 57°C for 1 min., then melt curve (0.11°C per sec from 57°C to 95°C). C_t_ values were calculated on the LighCycler software using absolute quantification 2^nd^ derivative max. For each plate, all reactions were performed in quadruplicate, representing technical replicates. The specificity of all primer-probe pairs was confirmed using genomic DNA from a mouse heterozygous for the insertion as a positive control and a wild-type littermate as a negative control. No signal was detected in negative control samples. The sensitivity of each primer-probe set to detect mosaicism for the insertion was ascertained by preparing “mosaicism standards”: genomic DNA from a heterozygous insertion-bearing mouse and a wild-type mouse was mixed at defined ratios (0.1% insertion prevalence to 90% insertion prevalence). Control assays to establish sensitivity and assays for mosaicism within tissues were always run alongside DNA from a heterozygous mouse (100% prevalence) and a wild-type littermate (0% prevalence).

C_t_ values obtained in each experiment were analyzed by averaging the four technical replicates for each L1 insertion reaction and corresponding RPP25 control reaction, and establishing the ΔC_t_ value for each sample for the L1 insertion relative to the RPP25 control reaction. The heterozygous control animal for each experiment was designated as 100% prevalence, and the ΔΔC_t_ values of all other samples were calculated relative to the heterozygous control to quantify percent prevalence of the insertion. Sensitivity control assays were performed once, and error represented as the standard deviation of four technical replicates. Assays to quantify mosaicism within tissues were performed three independent times (biological replicates), each consisting four technical replicates per sample, and error reported as the standard deviation of three biological replicates.

**References**

Chang YF, Lee-Chang JS, Panneerdoss S, MacLean JA, 2nd, Rao MK. 2011. Isolation of Sertoli, Leydig, and spermatogenic cells from the mouse testis. *BioTechniques* **51**(5): 341-342, 344.

Dombroski BA, Scott AF, Kazazian HH, Jr. 1993. Two additional potential retrotransposons isolated from a human L1 subfamily that contains an active retrotransposable element. *Proc Natl Acad Sci U S A* **90**(14): 6513-6517.

Freeman JD, Goodchild NL, Mager DL. 1994. A modified indicator gene for selection of retrotransposition events in mammalian cells. *BioTechniques* **17**(1): 46, 48-49, 52.

Goodier JL, Ostertag EM, Du K, Kazazian HH, Jr. 2001. A novel active L1 retrotransposon subfamily in the mouse. *Genome Res* **11**(10): 1677-1685.

Kopera HC, Larson PA, Moldovan JB, Richardson SR, Liu Y, Moran JV. 2016. LINE-1 Cultured Cell Retrotransposition Assay. *Methods Mol Biol* **1400**: 139-156.

Moran JV, Holmes SE, Naas TP, DeBerardinis RJ, Boeke JD, Kazazian HH, Jr. 1996. High frequency retrotransposition in cultured mammalian cells. *Cell* **87**(5): 917-927.

Naas TP, DeBerardinis RJ, Moran JV, Ostertag EM, Kingsmore SF, Seldin MF, Hayashizaki Y, Martin SL, Kazazian HH. 1998. An actively retrotransposing, novel subfamily of mouse L1 elements. *EMBO J* **17**(2): 590-597.

Sassaman DM, Dombroski BA, Moran JV, Kimberland ML, Naas TP, DeBerardinis RJ, Gabriel A, Swergold GD, Kazazian HH, Jr. 1997. Many human L1 elements are capable of retrotransposition. *Nat Genet* **16**(1): 37-43.

Wei W, Morrish TA, Alisch RS, Moran JV. 2000. A transient assay reveals that cultured human cells can accommodate multiple LINE-1 retrotransposition events. *Anal Biochem* **284**(2): 435-438.
