# Supplementary material for: Heritable L1 retrotransposition in the mouse primordial germline and early embryo
Source: Genome Res. 2017 Aug;27(8):1395–405. doi: 10.1101/gr.219022.116 (PMC5538555; doi:10.1101/gr.219022.116)
Supplement: Supplemental Material [file supp_gr.219022.116_Supplemental_fig_S4.pdf]

Supplemental Figure 4

Insertion #1

chr12 (qF1), +strand

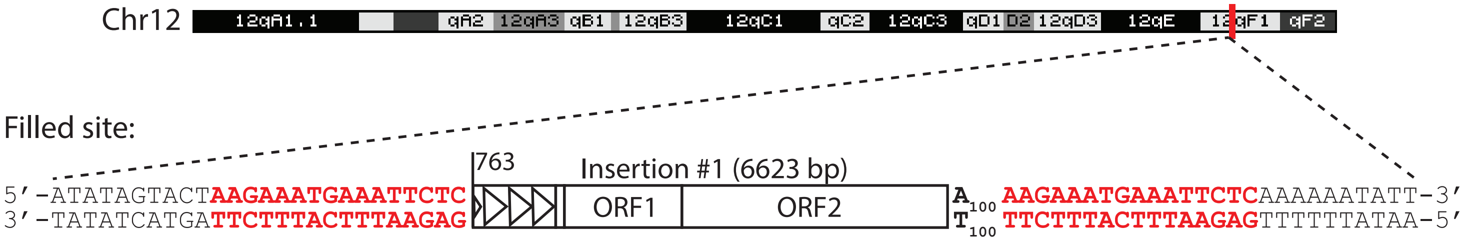

Empty site:

5′-AGGGAAATGGCGGAAATATAGTACT**AAGAAATGAAATTCTC**AAAAAATATTTAAAAAAAATCAGCA-3′
3′-TCCCTTTACCGCCTTTATATCATGA**TTCTTTACTTTAAGAG**TTTTTTATAAATTTTTTTTAGTCGT-5′

1st strand cleavage

L1 subfamily: Tf
Monomers: 3 + 77bp

TSD: 16 bp
(Chr12, 109324399 - 109324414)

EN motif: 5′-TCTT/AG-3′

Developmental origin: male (SRB) primordial germline

Insertion #2

chr2 (qE1), -strand

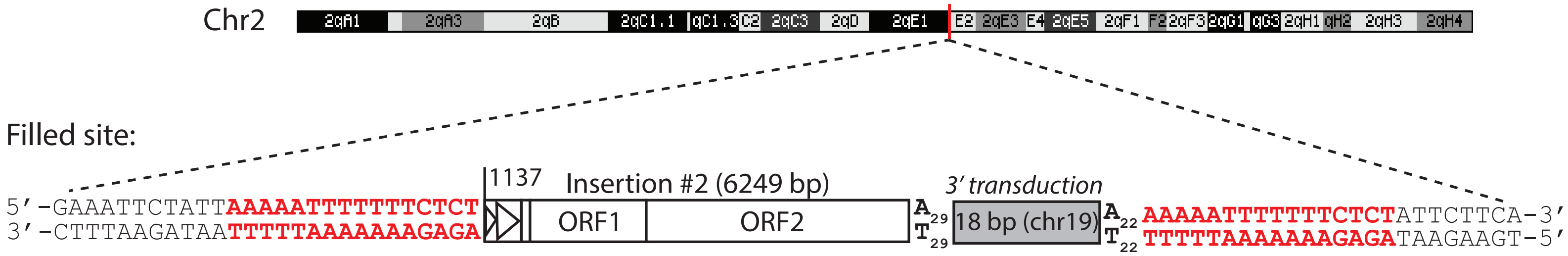

Empty site:

5′-AGGCCTTGATGTAGTTTGAAATTCTATT**AAAAATTTTTTTCTCT**ATTCTTCACTATATTTCATTGAGCAATG-3′
3′-TCCGGAACTACATCAAACTTTAAGATAA**TTTTTAAAAAAAGAGA**TAAGAAGTGATATAAAGTAACTCGTTAC-5′

1st strand cleavage

L1 subfamily: Tf
Monomers: 1 + 124bp

TSD: 16 bp
(Chr2: 101251997-101252012)

EN motif: 5′-TTTT/AA-3′

Developmental origin: Male (SRB) primordial germline

Supplemental Figure 4 (cont'd)

Insertion #3

chr10 (qB3), -strand

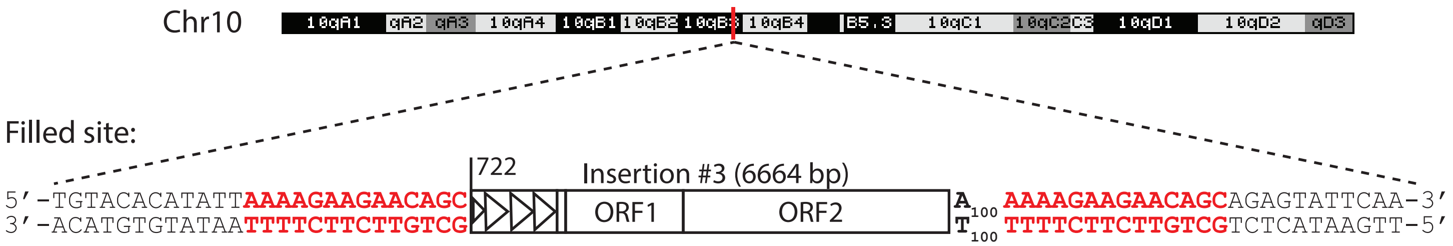

Empty site:

5'-TGTCATGAAGAATGTACACATATTAAAAGAAGAACAGCAGAGTATTCAAGGAGGCAGCAAA-3'
3'-ACAGTACTTCTTACATGTGTATAATTTTCTTCTTGTCGTCTCATAAGTTCCTCCGTCGTTT-5'

1st strand cleavage

L1 subfamily: Tf

Monomers: 3 + 118 bp

TSD: 14 bp
(chr10: 55,125,883-55,125,896)

EN motif: 5'-TTTT/AA-3'

Developmental origin: female (SRA) early embryo

Insertion #4

chr3 (qE3), +strand

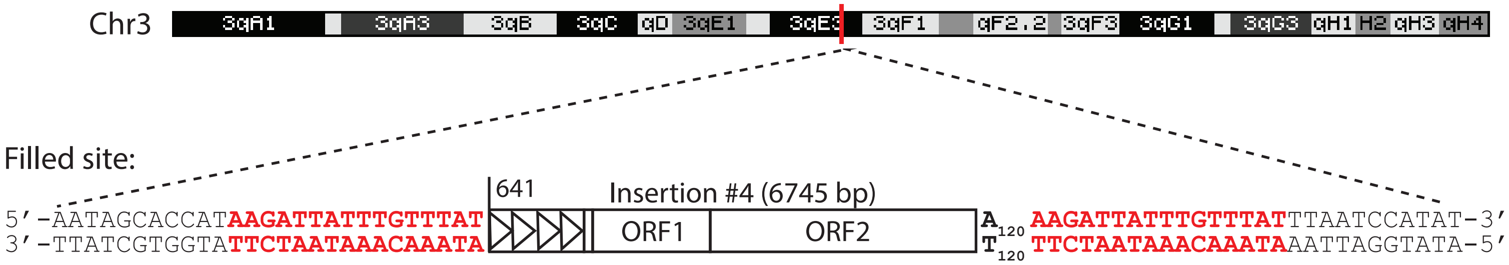

Empty site:

5'-ACCTTATAGACTTCTTAATAGCACCATAAGATTATTTGTTTATTTAATCCATATTTGCTTAATTTA-3'
3'-TGGAATATCTGAAGAATTATCGTGGTATTCTAATAAACAAATAAATTAGGTATAAACGAATTAAAT-5'

1st strand cleavage

L1 subfamily: Tf

Monomers: 4 minus 10 bp

TSD: 16 bp
(chr3: 81,424,430-81,424,445)

EN motif: 5'-TCTT/AT-3'

Developmental origin: Late germline (SRE or SRF)

Supplemental Figure 4 (cont'd)

Insertion #5

chr10 (qC1), +strand

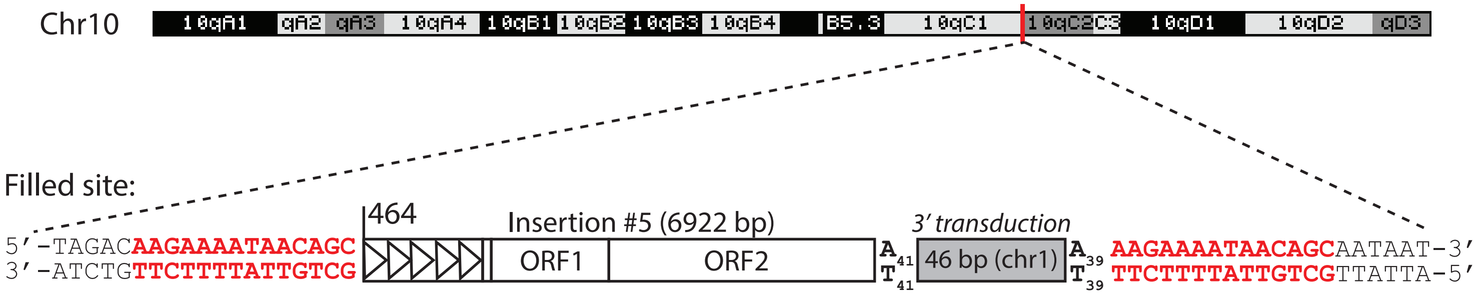

Empty site:

5′-GATTTTTGTAAGATATCTTAGTAGACAAGAAAATAACAGCAATAATTTATAAATGGAATCGTA-3′
3′-CTAAAAACATTCTATAGAATCATCTGTTCTTTTATTGTCGTTATTAAATATTTACCTTAGCAT-5′

↑
1st strand cleavage

L1 subfamily: Tf
Monomers: 5 minus 43 bp

TSD: 14 bp
(chr10: 89,133,398 - 89,133,411)

EN motif: 5′-TCTT/GT-3′

Developmental origin: Female (SRE) early embryo

Insertion #6

chr14 (qE2.3), -strand

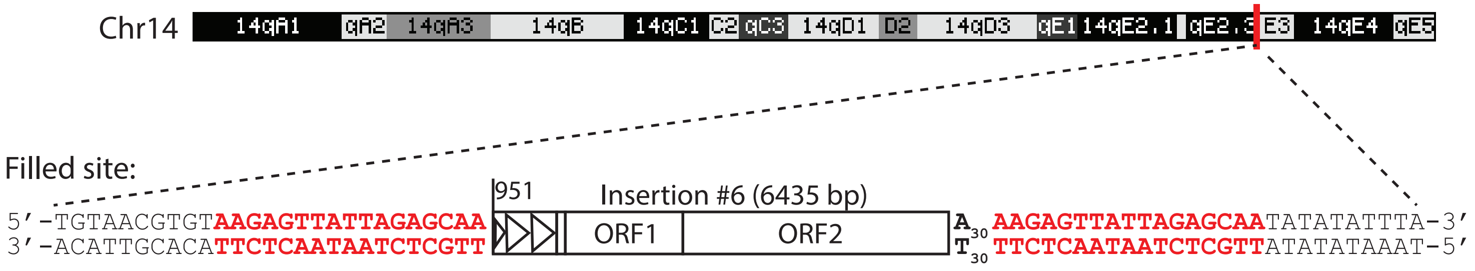

Empty site:

5′-CAGAATTTGAGTAACGTGTAACGTGTAAGAGTTATTAGAGCAATATATATTTATACCATCTTTAAGTT-3′
3′-GTCTTAAACTCATTGCACATTGCACATTCTCAATAATCTCGTTATATATAAATATGGTAGAAATTCAA-5′

↑
1st strand cleavage

L1 subfamily: Tf
Monomers: 2 + 100bp

TSD: 17 bp
(Chr14, 107,023,820 - 107,023,836)

EN motif: 5′-TTCT/AC-3′

Developmental origin: Female (SRCD10) early embryo

Supplemental Figure 4 (cont'd)

Insertion #7

chr18 (qE4), -strand

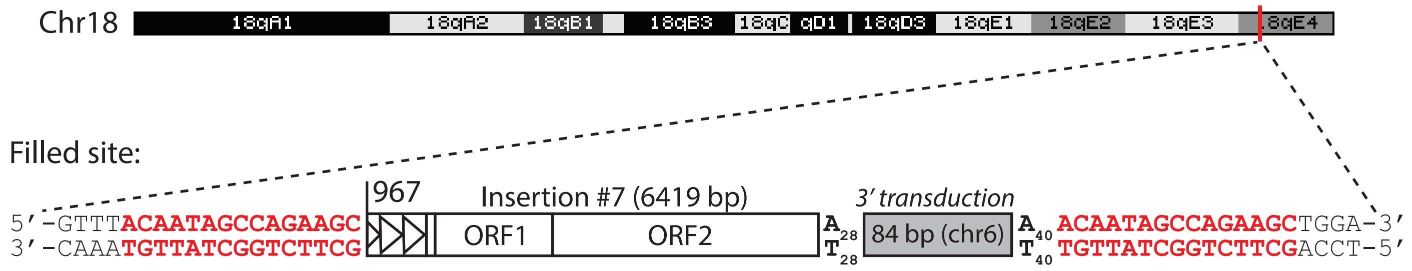

Empty site:

5'-ATTACGTTCATAGCAGCCTTGTTT**ACAATAGCCAGAAGC**TGGAAAGAACCCAGTTGTTTCTC-3'
3'-TAATGCAAGTATCGTCGGAACAAA**TGTTATCGGTCTTCG**ACCTTTCTTGGGTCAACAAAGAG-5'

1st strand cleavage

L1 subfamily: Tf
Monomers: 2 + 84bp

TSD: 15 bp
(Chr18: 85,067,455 - 85,067,469)

EN motif: 5'-TTGT/AA-3'

Developmental origin: Male (SRCD14) primordial germline

Insertion #8

chr17 (qC), +strand

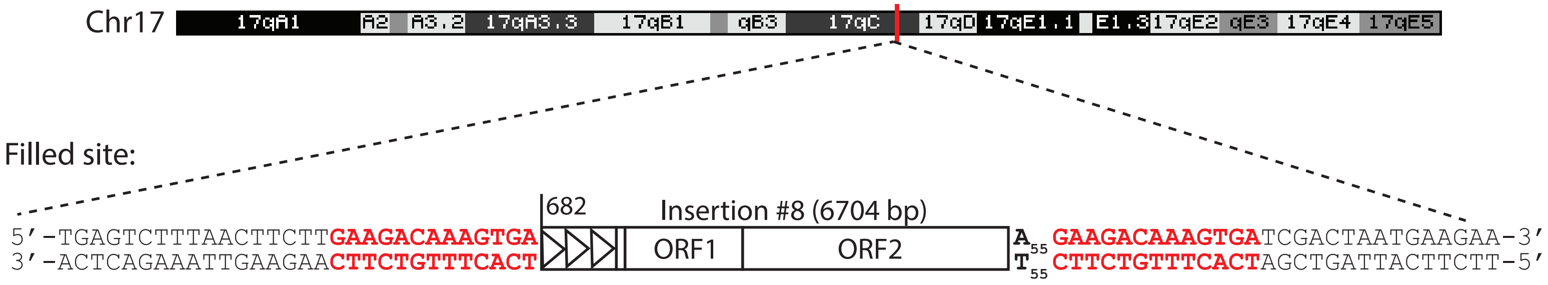

Empty site:

5'-TTGAATATCTTCTGAGTCTTTAACTTCTT**GAAGACAAAGTGA**TCGACTAATGAAGAAATGCCTTATTCT-3'
3'-AACTTATAGAAGACTCAGAAATTGAAGAA**CTTCTGTTTCACT**AGCTGATTACTTCTTTACGGAATAAGA-5'

1st strand cleavage

L1 subfamily: Tf
Monomers: 3 minus 52bp

TSD: 13 bp
(Chr17: 54,164,875- 54,164,887)

EN motif: 5'-CTTC/AA-3'

Developmental origin: Female (SREF17) early embryo

Supplemental Figure 4 (cont'd)

Insertion #9

chr2 (qC1.3), -strand

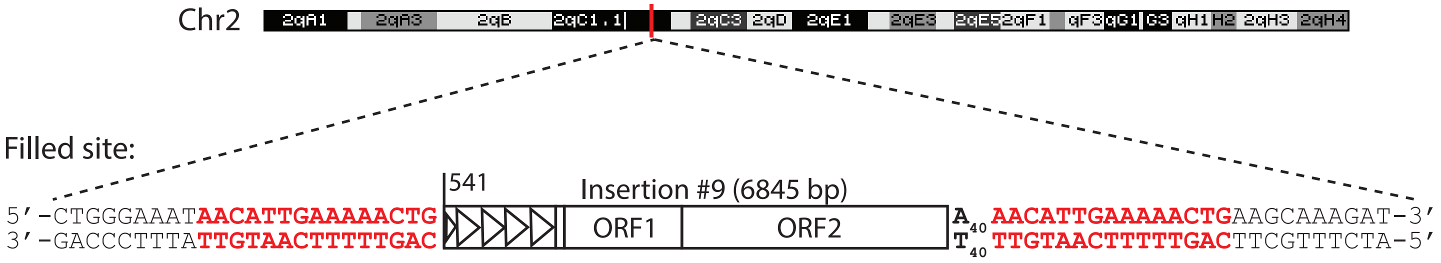

Empty site:

```
5'-CCGTTTAAGAAGAATAAACTGGGAAATAACATTGAAAAACTGAAGCAAAGATTTGCTTCCCTGCTTA-3'
3'-GGCAAATTCTTCTTATTTGACCCTTTATTGTAACTTTTTGACTTCGTTTCTAAACGAAGGGACGAAT-5'
```

↑ 1st strand cleavage

L1 subfamily: Tf
Monomers: 4 + 90bp

TSD: 15 bp
(Chr2: 65,072,088 - 65,072,102)

EN motif: 5'-TGTT/AT-3'

Developmental origin: Ambiguous, late germline? (Female SRCD11 or Male SREF19)

Insertion #10

chr3 (qA1), -strand

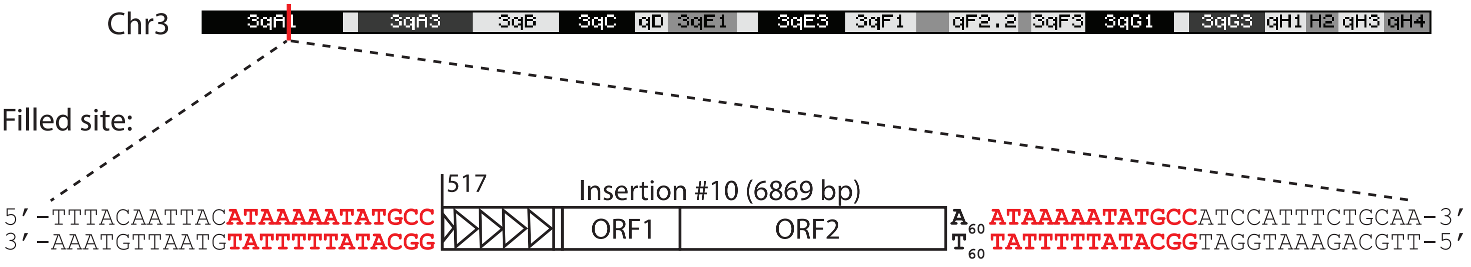

Empty site:

```
5'-TGTTGATTTCATCAGTATTTACAATTACATAAAAATATGCCATCCATTTCTGCAATAAATATTAATTA-3'
3'-ACAACTAAAGTAGTCATAAATGTTAATGTATTTTTATACGGTAGGTAAAGACGTTATTTATAATTAAT-5'
```

↑ 1st strand cleavage

L1 subfamily: Tf
Monomers: 4 + 114bp

TSD: 13 bp
(Chr3: 11,610,299 - 11,610,311)

EN motif: 5'-TTAT/GT-3'

Developmental origin: Male early embryo (F2-145)

Supplemental Figure 4 (cont'd)

Insertion #11

chr8 (qB3.1), +strand

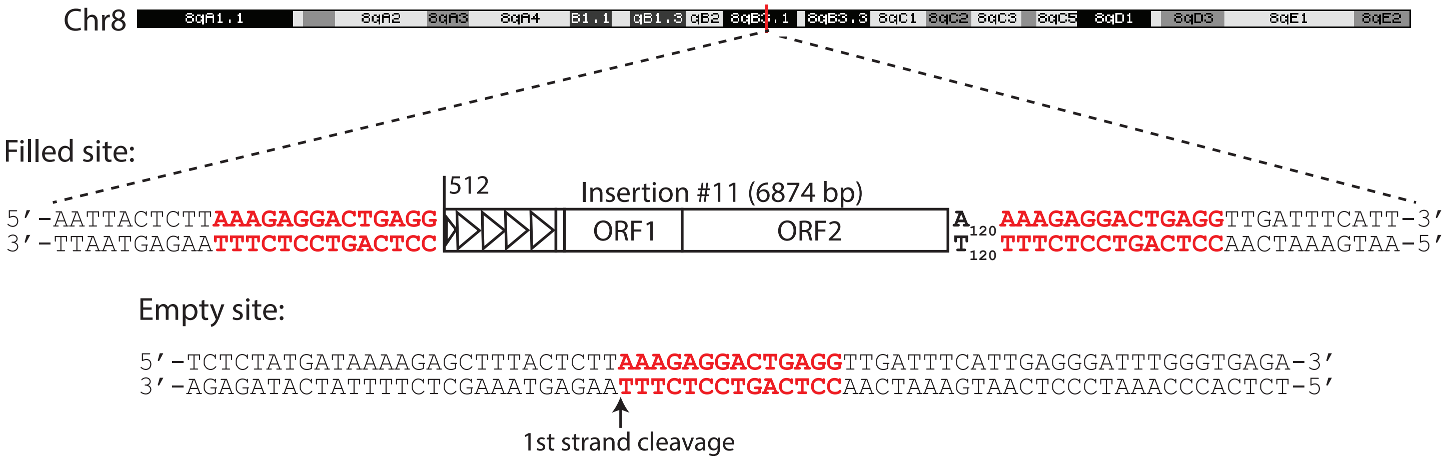

L1 subfamily: Tf

Monomers: 4 + 119bp

TSD: 14 bp

(Chr8: 63,990,917-63,990,930)

EN motif: 5'-TCTT/AA-3'

Developmental origin: Female (SRA) early embryo

Supplemental Figure S4. Structures of *de novo* L1 T_F insertions identified in this study.

De novo insertions are annotated as described for polymorphic insertions (Fig. S3), and the deduced developmental timing of each insertion is noted.
