# Supplementary material for: Heritable L1 retrotransposition in the mouse primordial germline and early embryo
Source: Genome Res. 2017 Aug;27(8):1395–405. doi: 10.1101/gr.219022.116 (PMC5538555; doi:10.1101/gr.219022.116)
Supplement: Supplemental Material [file supp_gr.219022.116_Supplemental_Table_5.pdf]

Supplemental Table 5: mRC-seq Read Counts for Mosaic Insertions

	% Prevalence: insertion #3	Reads: insertion #3	Reference L1 5’ end sequencing depth (fold)
**SRA Liver**	7.5	0	34.79
**SRA Brain**	3.7	0	37.28
**SRA Muscle**	4.2	0	21.62
**SRA Right Ovary**	27.5	1	26.89
**SRA Left Ovary**	9.1	0	27.33
	**% Prevalence: insertion #5**	**Reads: insertion #5**	**Reference L1 5’ end sequencing depth (fold)**
**SRE Liver**	0.2	0	53.59
**SRE Brain**	0.6	1	27.38
**SRE Heart**	0.2	0	27.60
**SRE Right Ovary**	1.5	0	30.16
**SRE Left Ovary**	0.4	0	31.14
	**% Prevalence: insertion #7**	**Reads: insertion #7**	**Reference L1 5’ end sequencing depth (fold)**
**SRCD14 Right Testicle**	3.6	2	277.06
**SRCD14 Left Testicle**	11.5	13	238.56

Supplemental Table 5. mRC-seq read counts for mosaic insertions. For each insertion (#3, #5, #7) where the tissues of the appropriate mosaic animal were assayed individually by mRC-seq, the percent prevalence of the insertion and the mRC-seq read count are shown. As a reference for sequence capture efficiency and sequencing depth for each library, sequencing depth achieved for reference L1 5' ends is shown. For insertion #6 in mouse SRCD10, the tissues of mouse SRCD10 were not analyzed individually by mRC-seq, so read counts and reference L1 sequencing depth are denoted as n/a. Full statistics for each library can be found in Supplemental Table 1.
