# Supplementary material for: Heritable L1 retrotransposition in the mouse primordial germline and early embryo
Source: Genome Res. 2017 Aug;27(8):1395–405. doi: 10.1101/gr.219022.116 (PMC5538555; doi:10.1101/gr.219022.116)
Supplement: Supplemental Material [file supp_gr.219022.116_Supplemental_fig_S2.pdf]

Supplemental Figure 2

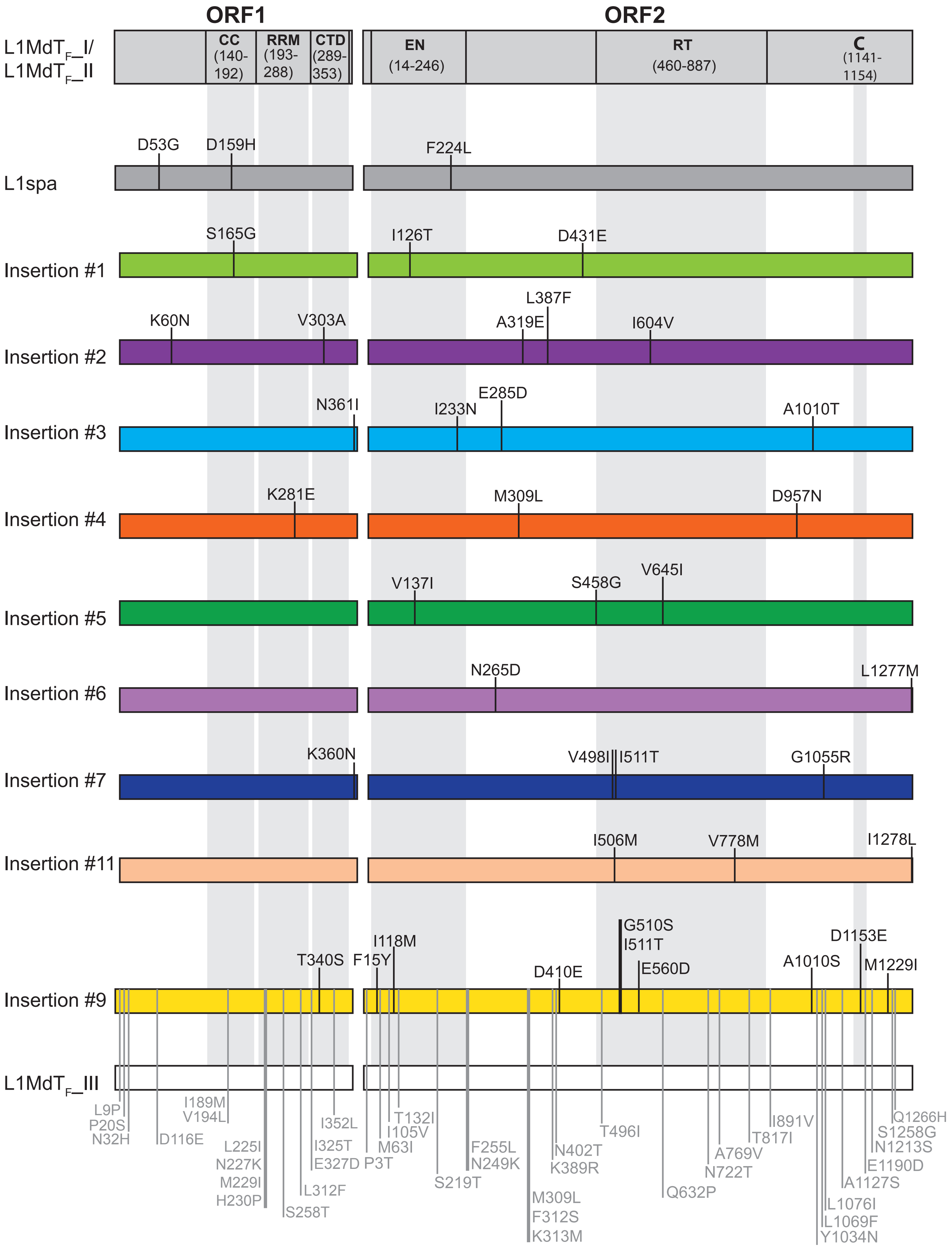

Supplemental Figure S2. Sequence comparison of *de novo* L1 insertions

Amino acid substitutions in ORF1 and ORF2 of $L1_{spa}$ and 9 fully sequenced *de novo* L1 insertions from this study, compared to the amino acid sequence of the $L1MdT_F$_I and $L1MdT_F$_II consensus sequences(Sookdeo et al. 2013). The amino acid sequences of $L1MdT_F$_I and $L1MdT_F$_II are identical to one another. The locations of functional domains in ORF1 and ORF2 are shown: CC = coiled-coil, RRM = RNA recognition motif, CTD = C-terminal domain, EN = endonuclease, RT = reverse transcriptase, C = cysteine-rich motif. Insertion #9 closely resembles the $L1MdT_F$_III consensus. Amino acid substitutions relative to $L1MdT_F$_I/$L1MdT_F$_II that are shared by insertion #9 and the $L1MdT_F$_III consensus are indicated below the $L1MdT_F$_III schematic, in grey. Amino acid substitutions unique to insertion #9 are shown above the insertion #9 schematic in black.
