# Supplementary material for: Heritable L1 retrotransposition in the mouse primordial germline and early embryo
Source: Genome Res. 2017 Aug;27(8):1395–405. doi: 10.1101/gr.219022.116 (PMC5538555; doi:10.1101/gr.219022.116)
Supplement: Supplemental Material [file supp_gr.219022.116_Supplemental_fig_S9.pdf]

Supplemental Figure 9

A.

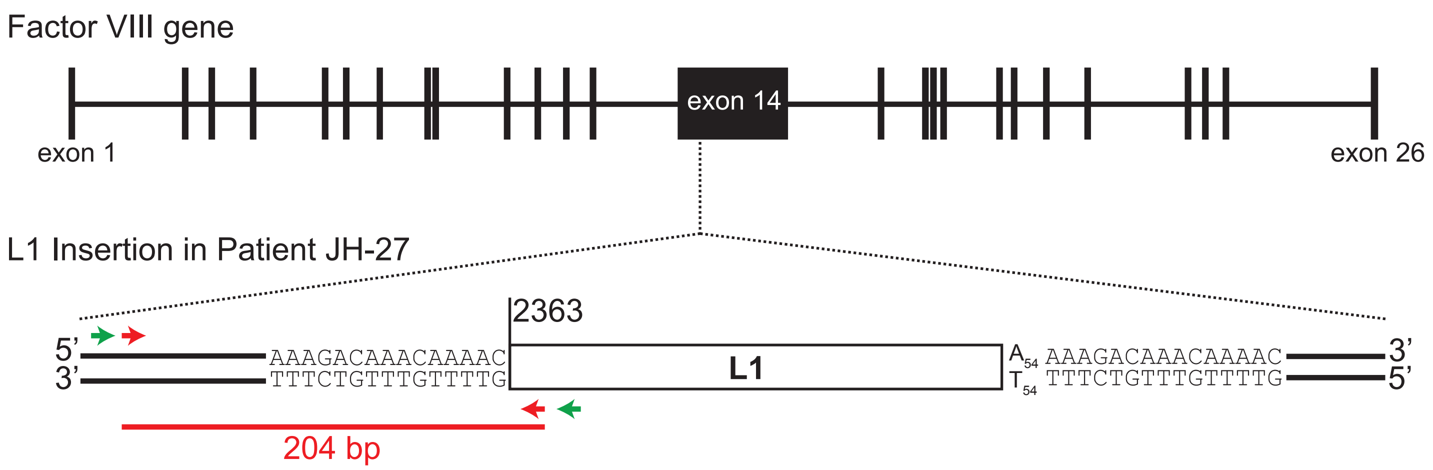

B.

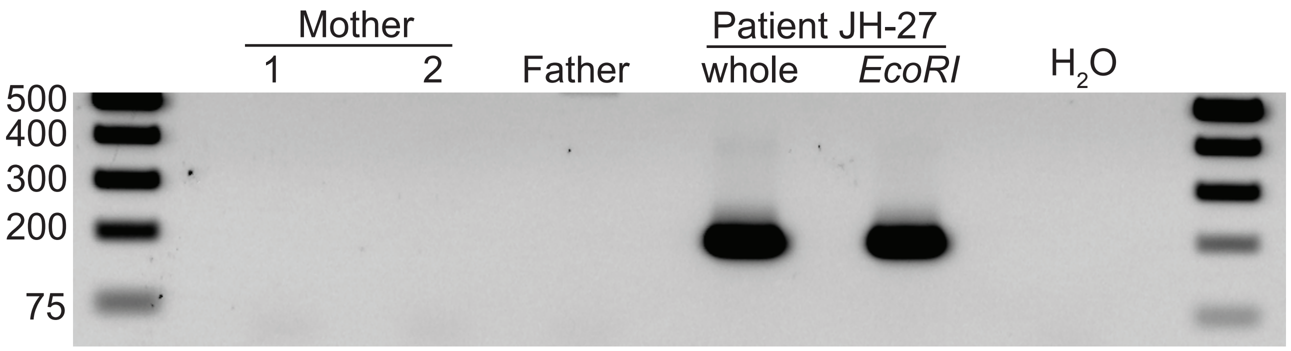

Supplemental Figure S9. PCR genotyping of the JH-27 human L1 insertion.

A. The relative position of the JH-27 insertion within exon 14 of the Factor VIII gene is depicted. Structural features of the insertion, including target-site duplications, poly(A) tail, and 5' truncation point are indicated. The relative positions of the nested PCR primers used for genotyping and the expected PCR fragment are shown; inner primers are indicated in red; outer primers are shown in green.

B. PCR genotyping of JH-27 insertion. From left, PCR templates comprised two samples of maternal blood gDNA, paternal blood gDNA, patient JH-27 blood gDNA, a sample of patient JH-27 blood gDNA that had previously been digested with EcoRI, and water (no template control).
