# Supplementary material for: Heritable L1 retrotransposition in the mouse primordial germline and early embryo
Source: Genome Res. 2017 Aug;27(8):1395–405. doi: 10.1101/gr.219022.116 (PMC5538555; doi:10.1101/gr.219022.116)
Supplement: Supplemental Material [file supp_gr.219022.116_Supplemental_Table_3.pdf]

Supplemental Table 3: Polymorphic Retroelement Insertions

Insertion	Location	T_F Monomers	Cleavage	TSD (bp)	PolyA (bp)	Fixed?
Poly_B1_1	16q	n/a	TTTT/GA	13	~39	no
Poly_B1_2	4q	n/a	CTTT/AA	12	~51	no
Poly_B2_1	Xq	n/a	TTTT/AT	15	~63	no
Poly_B2_2	5q	n/a	TTTT/AA	15	~100	yes
Poly_B2_3	4q	n/a	TTTT/AT	2	0	yes
Poly_B2_4	9q	n/a	TTTT/GA	10	~87	yes
Poly_L1T_F_1	15q	3.5	CTTT/AA	12	~100	no
Poly_L1T_F_3	6q	2.5	TCTT/AT	14	~86*	no
Poly_L1T_F_4	17q	2.5	TTTC/AA	16	~90*	no
Poly_L1T_F_5	1q	4.5	TTTA/AA	15	~50	no
Poly_L1T_F_10	10q	5.5	TTTC/AA	14	~81	yes
Poly_L1T_F_11	15q	2.5	TTTT/GG	20	~110	yes
Poly_L1T_F_12	5q	4.5	TTAT/AG	13	~21	yes
Poly_L1T_F_14	11q	3.5	TCTT/AA	13	~44	no
Poly_L1T_F_15	12q	6.5	TTCT/GT	16	~54	no
Poly_L1T_F_18	4q	3	TTTT/CA	17	~30	yes
Poly_L1T_F_19	7q	3	ATTT/AA	16	~21	yes

Supplemental Table 3. Characteristics of polymorphic retrotransposon insertions characterized in this study. All L1 insertions shown are L1 T_F elements; T_F monomers = the number of repetitive promoter units contained by each insertion. Cleavage indicates the L1 endonuclease cleavage motif, shown 5' to 3'. TSD = target-site duplication length, Poly(A) = poly(A) tract length. Poly(A) tract lengths were determined by Sanger sequencing and should be regarded as estimates. Insertions denoted as fixed were detected in all animals assessed by mRC-seq in this study; those denoted as not fixed were differentially present/absent among animals. Insertions marked with an asterisk contain a 3' transduction (see Fig. S3).
