# Supplementary material for: Heritable L1 retrotransposition in the mouse primordial germline and early embryo
Source: Genome Res. 2017 Aug;27(8):1395–405. doi: 10.1101/gr.219022.116 (PMC5538555; doi:10.1101/gr.219022.116)
Supplement: Supplemental Material [file supp_gr.219022.116_Supplemental_fig_S7.pdf]

Supplemental Figure 7

A.

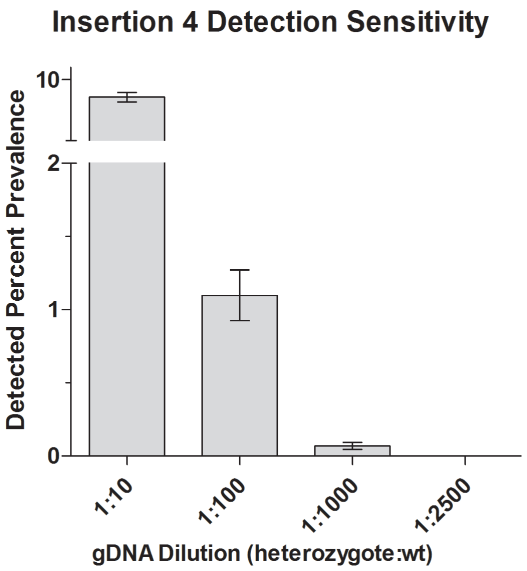

B.

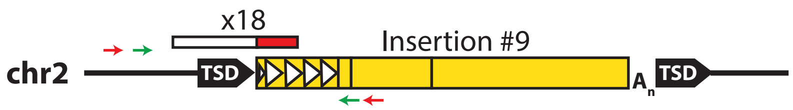

C.

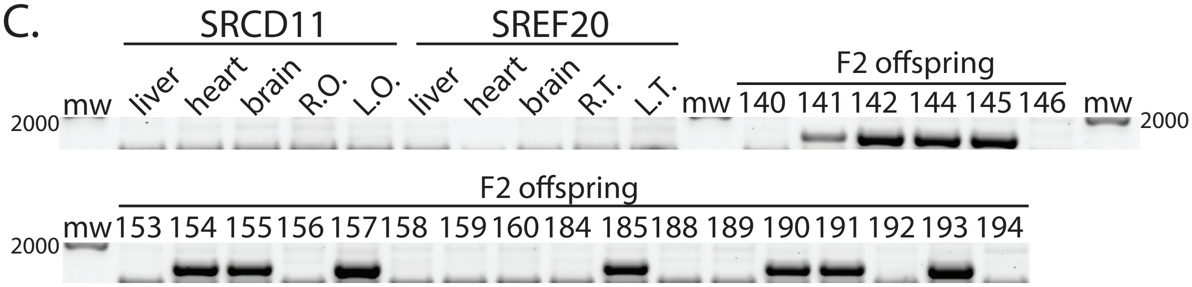

D.

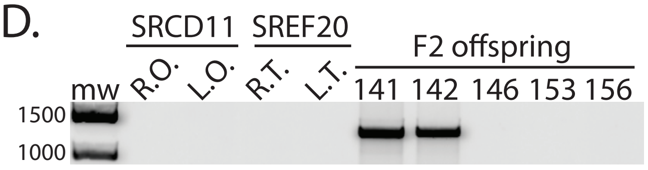

Supplemental Figure S7. Insertions arising in the late germline

A. Assay for the lower limit of sensitivity for insertion #4 qPCR. The x-axis indicates the ratio of genomic DNA (heterozygous mouse: mouse lacking Insertion #4) used as a template, the y-axis indicates percent prevalence detected relative to a heterozygous animal. Data are reported as the mean and standard deviation of four technical replicates per reaction.

B. Schematic of insertion #9, annotated as in Fig. 2A. The position of genotyping PCR primers for the 5' junction is shown; red arrows indicate outer nested primers and green arrows indicate inner nested primers.

C. Genotyping panel for Insertion #9 among the somatic tissues and gonads of SRCD11 and SREF20, and the F2 offspring of SRCD11 and SREF20. For this panel, PCR was not nested and only the outer (red) primers were used.

D. Nested PCR to detect Insertion #9 in the gonads of SRCD11 and SREF20. The fully nested primers depicted on the schematic in panel F were used. F2 offspring containing the insertion (141 and 142) and lacking the insertion (146, 153, and 156) served as positive and negative controls.
