# Supplementary material for: Heritable L1 retrotransposition in the mouse primordial germline and early embryo
Source: Genome Res. 2017 Aug;27(8):1395–405. doi: 10.1101/gr.219022.116 (PMC5538555; doi:10.1101/gr.219022.116)
Supplement: Supplemental Material [file supp_gr.219022.116_Supplemental_fig_S5.pdf]

Supplemental Figure 5

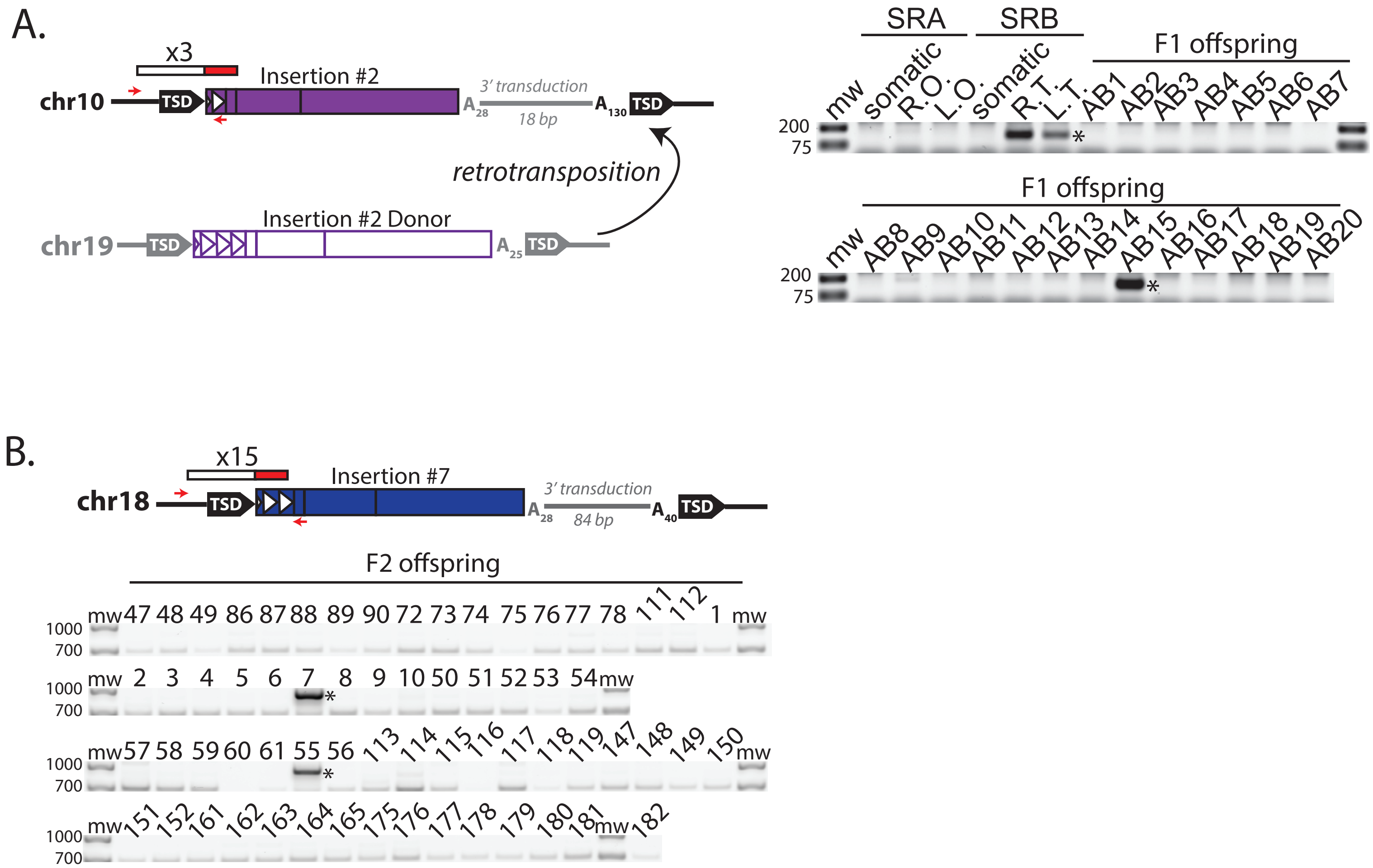

Supplemental Figure S5. Insertions arising in primordial germ cells.

A. Left: schematic of insertion #2 on Chr10 and its putative donor element on Chr19. Structural features of the L1 insertion and position of mRC-seq reads are depicted as in Fig. 2A. Red arrows indicate the position of validation PCR primers used to genotype mice for the insertion. Right: Genotyping panel consisting of parental mice SRA and SRB, and their F1 offspring. For parental mice, “somatic” indicates a mixture of genomic DNA from liver, brain, and skeletal muscle. R.O. and L.O. = right ovary and left ovary; R.T. and L.T. = right testicle and left testicle. For F1 offspring SRAB1-SRAB9, genotyping was performed on gDNA extracted from liver. F1 offspring 10-20 were harvested as embryos, and genotyping was performed on whole embryo gDNA.

B. Above: Schematic of insertion #7 as shown in Fig. 2C. Red arrows indicate the position of 5' junction validation PCR primers used to genotype the insertion. Below: Genotyping panel consisting of the 65 F2 offspring of SRCD14.
