# Supplementary material for: Heritable L1 retrotransposition in the mouse primordial germline and early embryo
Source: Genome Res. 2017 Aug;27(8):1395–405. doi: 10.1101/gr.219022.116 (PMC5538555; doi:10.1101/gr.219022.116)
Supplement: Supplemental Material [file supp_gr.219022.116_Supplemental_fig_S3.pdf]

Supplemental Figure 3

Poly_L1Tf_1

chr15 (qB3.3), +strand

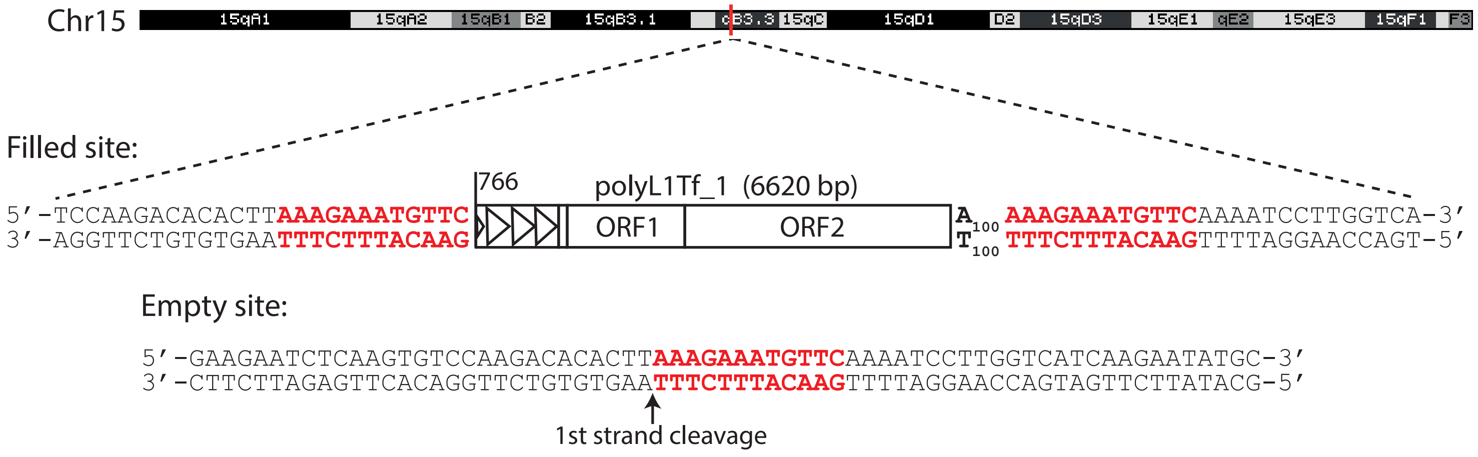

L1 subfamily: Tf

Monomers: 3 + 73 bp

TSD: 12 bp

(Chr15, 46,188,029 - 46,188,040)

EN motif: 5'-CTTT/AA-3'

Poly_L1Tf_3

chr6 (qE2), +strand

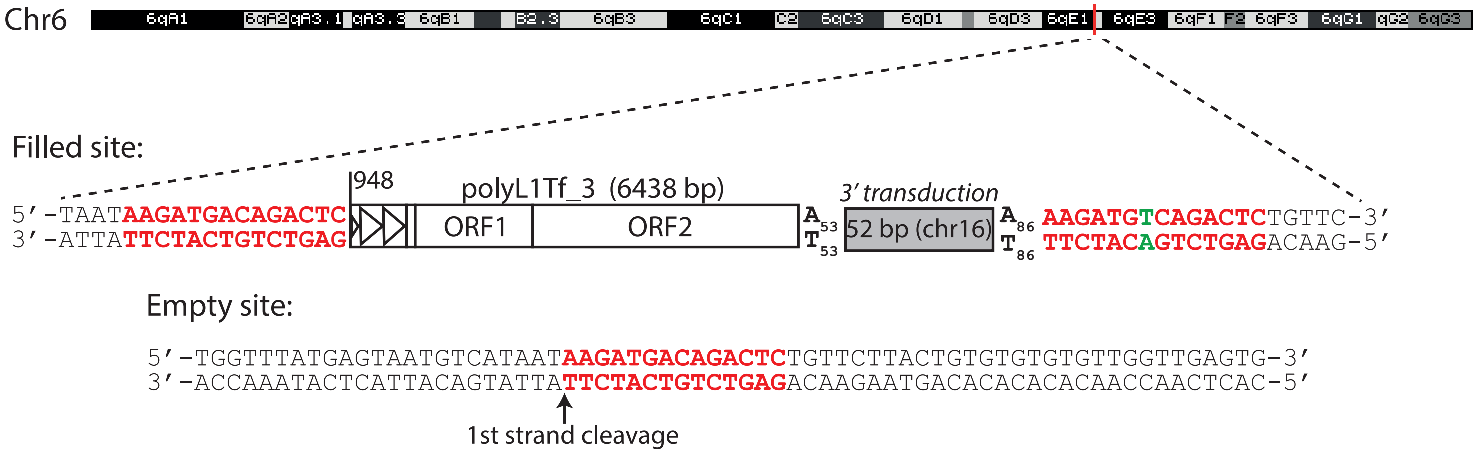

L1 subfamily: Tf

Monomers: 2 + 102 bp

TSD: 14 bp

(Chr6, 108,844,479 - 108,844,492)

EN motif: 5'-TCTT/AT-3'

Supplemental Figure 3 (cont'd)

Poly_L1Tf_4

chr17 (qB2), +strand

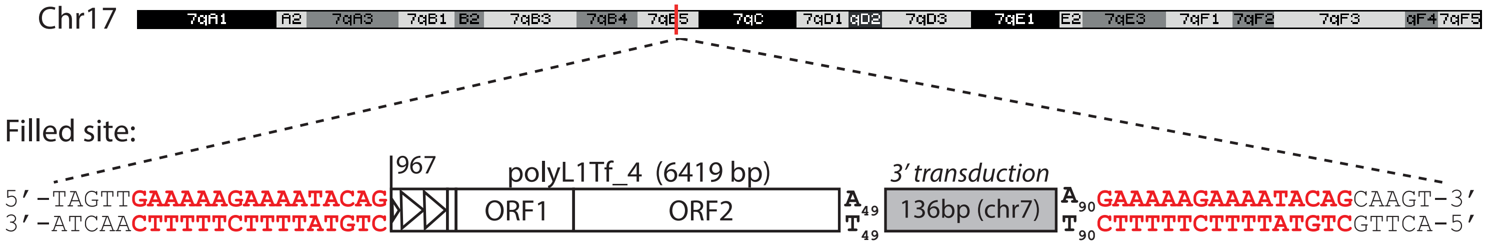

Empty site:

5'-ATTTTAAATTAAATGGGGATATAGTTGAAAAAGAAAATACAGCAAGTTGGTAATCTATAAATGAAATA-3'
3'-TAAAATTTAATTTACCCCTATATCAACTTTTTCTTTTATGTCGTTCAACCATTAGATATTTACTTTAT-5'

1st strand cleavage

L1 subfamily: Tf
Monomers: 2 + 83 bp

TSD: 16 bp
(Chr17; 41,334,114 - 41,334,129)

EN motif: 5'-TTTC/AA-3'

Poly_L1Tf_5

chr1 (qE2.1), -strand

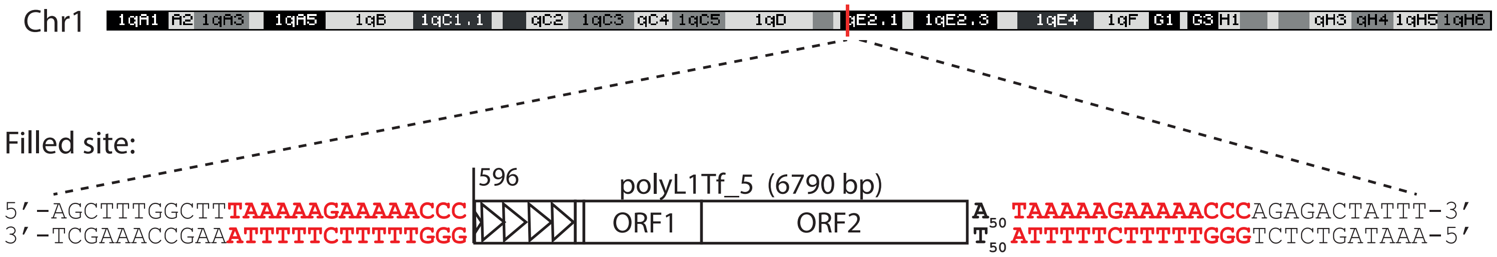

Empty site:

5'-TTTGCAAGTAGGCTTAGCTTTGGCTTTAAAAAGAAAAACCCAGAGACTATTTTGGAAAGGCTTAGTT-3'
3'-AAACGTTCATCCGAATCGAAACCGAAATTTTTCTTTTTGGGTCTCTGATAAAACCTTTCCGAATCAA-5'

1st strand cleavage

L1 subfamily: Tf
Monomers: 4 + 34 bp

TSD: 15 bp
(Chr1, 104,818,661 - 104,818,675)

EN motif: 5'-TTTA/AA-3'

Supplemental Figure 3 (cont'd)

Poly_L1Tf_10

chr10 (qB1), +strand

Chr10 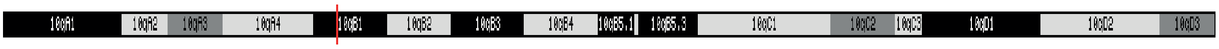

Filled site:

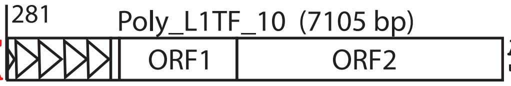

5'-AACTTATCCTT**GAAATATAAAAGCT** [L1] A_{81} **GAAATATAAAAGCT**ATTTCCATTAAA-3'
3'-TTGAATAGGAA**CTTTATATTTTCGA** [L1] T_{81} **CTTTATATTTTCGA**TAAAGGTAATTT-5'

Empty site:

5'-AGTCAAAGACAACATTAAACTTATCCTT**GAAATATAAAAGCT**ATTTCCATTAAAACCATGAACAAGAATTTG-3'
3'-TCAGTTTCTGTTGTAATTTGAATAGGAA**CTTTATATTTTCGA**TAAAGGTAATTTTGGTACTTGTTCTTAAAC-5'

↑ 1st strand cleavage

L1 subfamily: Tf
Monomers: 4 + 96 bp

TSD: 14 bp
(Chr10, 36,022,472 - 36,022,486)

EN motif: 5'-TTTC/AA-3'

polyL1Tf_11

chr15 (qB3.1), +strand

Chr15 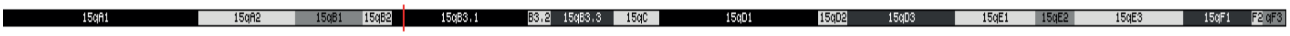

Filled site:

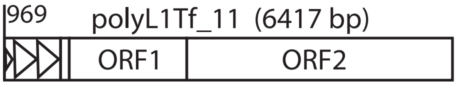

5'-TTCTCCC**AAAAGATCTTGTAGACAGGA** [L1] A_{100} **AAAAGATCTTGTAGACAGGA**TAAATTT-3'
3'-AAGAGGG**TTTTCTAGAACATCTGTCCT** [L1] T_{100} **TTTTCTAGAACATCTGTCCT**ATTTAAA-5'

Empty site:

5'-AAACTATGCTTCATCTTTCTCCC**AAAAGATCTTGTAGACAGGA**TAAATTTTGGGTAGAAGTTTTGT-3'
3'-TTTGATACGAAGTAGAAAGAGGG**TTTTCTAGAACATCTGTCCT**ATTTAAAACCCATCTTCAAAACA-5'

↑ 1st strand cleavage

L1 subfamily: Tf
Monomers: 2 + 81 bp

TSD: 20 bp
(Chr15, 45,572,157 - 45,572,177)

EN motif: 5'-TTTT/GG-3'

Supplemental Figure 3 (cont'd)

Poly_L1Tf_12

chr5 (qC3.1), +strand

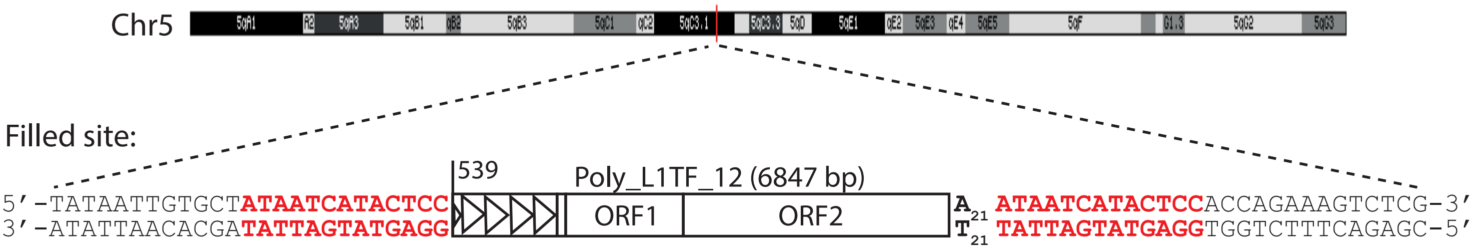

Empty site:

5'-TAGAAGAATTCCACTCTTTATAATTGTGCTATAATCATACTCCACCAGAAAGTCTCGAAATTAGTTC-3'
3'-ATCTTCTTAAGGTGAGAAATATTAACACGATATTAGTATGAGGTGGTCTTTCAGAGCTTTAATCAAG-5'

1st strand cleavage

L1 subfamily: Tf
Monomers: 4 + 61 bp

TSD: 13 bp
(Chr5, 69,153,918 - 69,153,936)

EN motif: 5'-TTAT/AG-3'

polyL1Tf_14

chr11 (qD), +strand

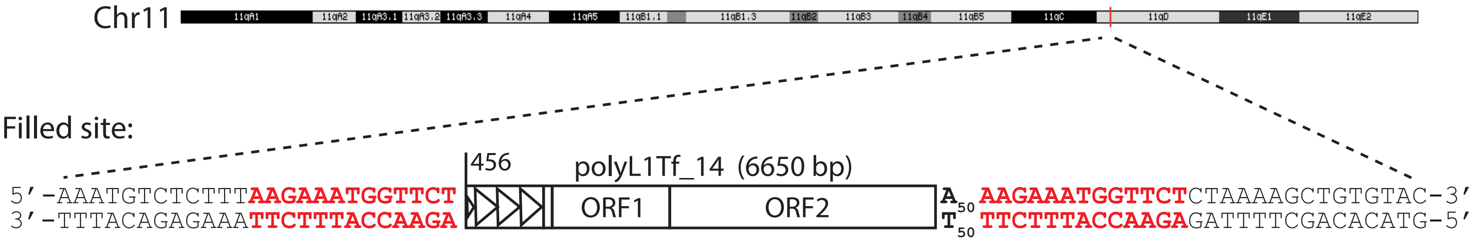

Empty site:

5'-TGATTGTTGGGAGGAAAATGTCTCTTTAAGAAATGGTTCTCTAAAAGCTGTGTACTTATACCTAGAA-3'
3'-ACTAACAACCCTCCTTTTACAGAGAAATTCTTTACCAAGAGATTTTCGACACATGAATATGGATCTT-5'

1st strand cleavage

L1 subfamily: Tf
Monomers: 3 + 101 bp

TSD: 13 bp
(Chr11, 91,759,570 - 91,759,583)

EN motif: 5'-TCTT/AA-3'

Supplemental Figure 3 (cont'd)

polyL1Tf_15

chr12 (qC1), +strand

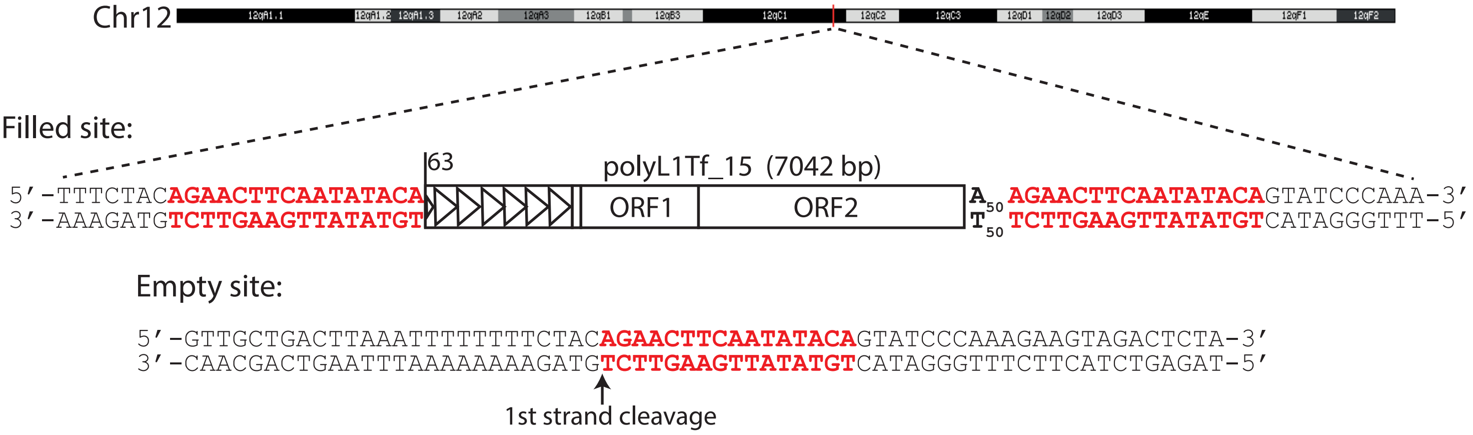

L1 subfamily: Tf

Monomers: 6 + 78 bp

TSD: 16 bp
(Chr12, 64,779,624 - 64,779,640)

EN motif: 5′-TTCT/GT-3′

Poly_L1Tf_18

chr4 (qE1), -strand

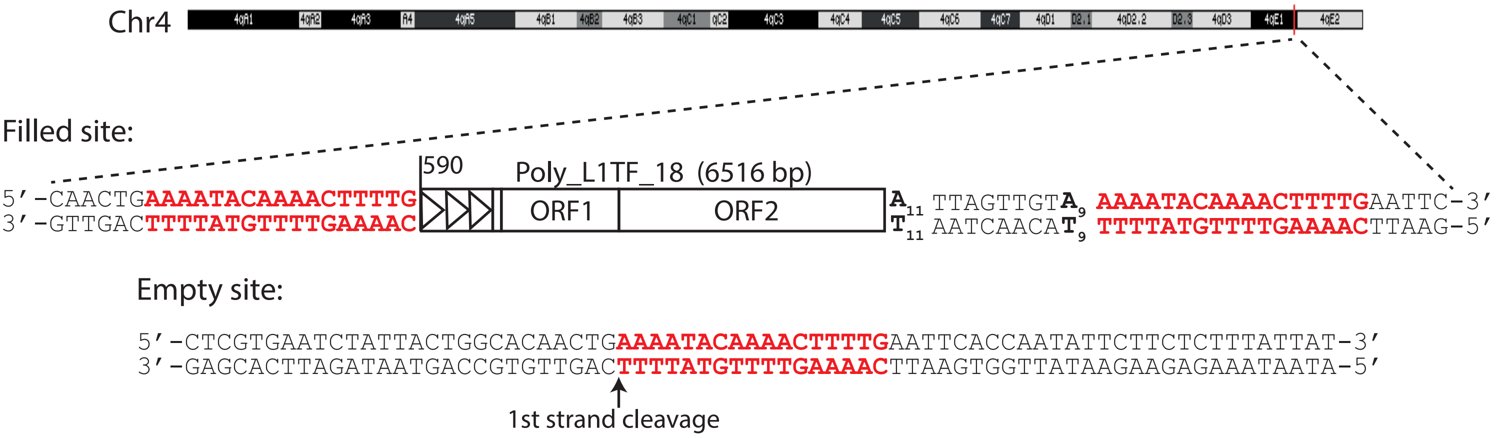

L1 subfamily: Tf

Monomers: 3

TSD: 17 bp
(Chr4, 147,497,685 - 147,497,704)

EN motif: 5′-TTTT/CA-3′

Supplemental Figure 3 (cont'd)

Poly_L1Tf_19

chr7 (qC), +strand

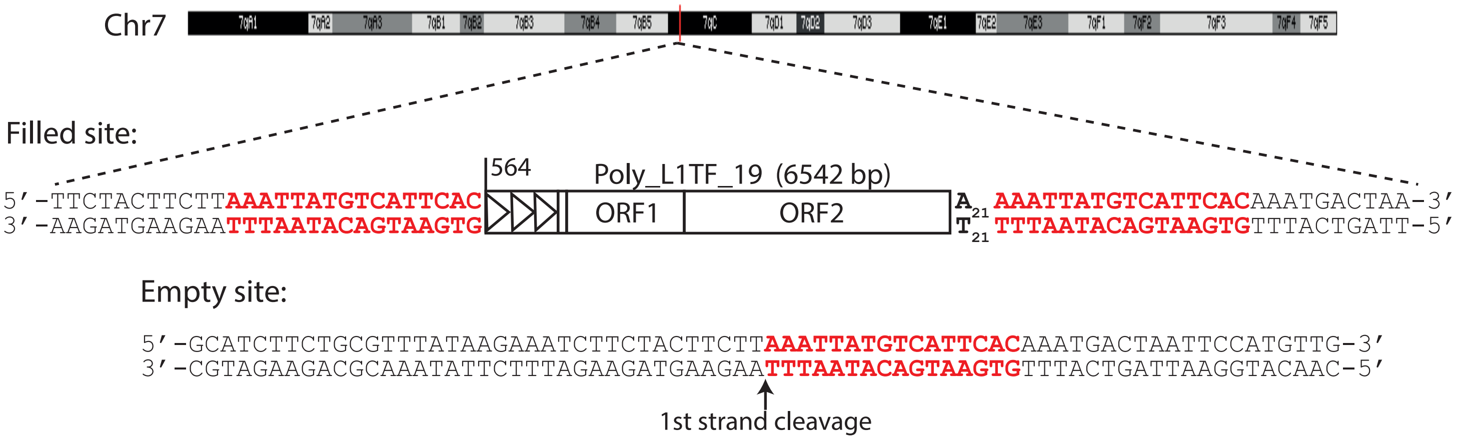

L1 subfamily: Tf

Monomers: 3

TSD: 16 bp

(Chr7, 62,347,340 - 62,347,358)

EN motif: 5'-ATTT/AA-3'

Supplemental Figure 3 (cont'd)

Poly_B1_1

chr16 (qC3.3), +strand

Chr16 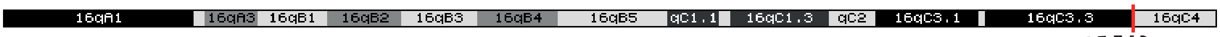

Filled site:

0 polyB1_1 (147 bp)

```
5'-CTGAAGTGAGGTCCTGTCTCAAAACTACTGGAG[          ]A39 AAAACTACTGGAGGATCTGGGATATAGTGTAG-3'
3'-GACTTCACTCCAGGACAGAGTTTTGATGACCTC[          ]T39 TTTTGATGACCTCCTAGACCCTATATCACATC-5'
```

Empty site:

```
5'-ATCAAGCTGAAGTGAGGTCCTGTCTCAAAACTACTGGAGGATCTGGGATATAGTGTAGTAGTAGT-3'
3'-TAGTTCGACTTCACTCCAGGACAGAGTTTTGATGACCTCCTAGACCCTATATCACATCATCATCA-5'
```

1st strand cleavage

SINE family: B1

TSD: 13 bp
(Chr16, 91599058 - 91599070)

EN motif: 5'-TTTT/GA-3'

Poly_B1_2

chr4 (qE1), -strand

Chr4 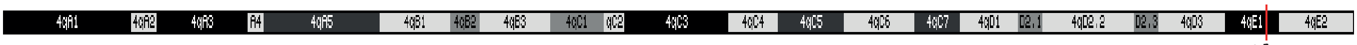

Filled site:

0 Poly_B1_2 (147 bp)

```
5'-GGGAGACAGATTATCAGACTTAAAGAAAAGGTA[          ]A51 AAAGAAAAGGTAGAGGAGGGGGTTACCTAAGT-3'
3'-CCCTCTGTCTAATAGTATGAATTTCTTTTCCAT[          ]T51 TTTCTTTTCCATCTCCTCCCCCAATGGATTCA-5'
```

Empty site:

```
5'-AAACTGTGGGAGACAGATTATCAGACTTAAAGAAAAGGTAGAGGAGGGGGTTACCTAAGTCAAA-3'
3'-TTTGACACCCTCTGTCTAATAGTCTGAATTTCTTTTCCATCTCCTCCCCCAATGGATTCAGTTT-5'
```

1st strand cleavage

SINE subfamily: B1

TSD: 12 bp
(Chr4, 146,430,138 - 146,430,150)

EN motif: 5'-CTTT/AA-3'

Poly_B2_1

chrX (qA4), +strand

ChrX 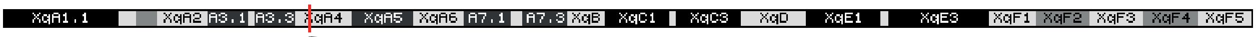

Filled site:

1 polyB2_1 (193 bp)

```
5'-AGCTTGTTTCACATATAAAAGACTGATACTC[          ]A63 AAAAGACTGATACTCACCTGTGCTTCTACTT-3'
3'-TCGAACAAAGTGTATATTTTCTGACTATGAG[          ]T63 TTTTCTGACTATGAGTGGACACGAAGATGAA-5'
```

Empty site:

```
5'-CACAGCTGCAAGCTTGTTTCACATATAAAAGACTGATACTCACCTGTGCTTCTACTTGTCTTTAAAA-3'
3'-GTGTCGACGTTCGAACAAAGTGTATATTTTCTGACTATGAGTGGACACGAAGATGAACAGAAATTTT-5'
```

1st strand cleavage

SINE family: B2

TSD: 15 bp
(Chr5, 41,903,727-41,903,741)

EN motif: 5'-TTTT/AT-3'

Supplemental Figure 3 (cont'd)

Poly_B2_2

chr5 (qE3), +strand

Chr5 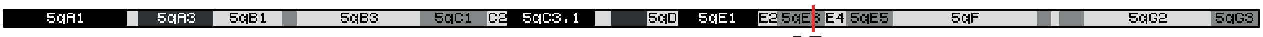

Filled site:

0 polyB2_2 (193 bp)

5′-CATGGACAGATCTTTT**AAAACATGACCATCC** [polyB2_2] A_{100} **AAAACATGACCATCC**AAAATAGAAGCAAGAT-3′
3′-GTACCTGTCTAGAAAA**TTTTGTACTGGTAGG** [polyB2_2] T_{100} **TTTTGTACTGGTAGG**TTTTATCTTCGTTCTA-5′

Empty site:

5′-ATATTGAAGACATGGACAGATCTTTT**AAAACATGACCATCC**AAAATAGAAGCAAGATGTTAGGGAGC-3′
3′-TATAACTTCTGTACCTGTCTAGAAAA**TTTTGTACTGGTAGG**TTTTATCTTCGTTCTACAATCCCTCG-5′

↑ 1st strand cleavage

SINE subfamily: B2

TSD: 15 bp
(Chr5, 98060042 - 98060056)

EN motif: 5′-TTTT/AA-3′

Poly_B2_3

chr4 (qE1), +strand

Chr4 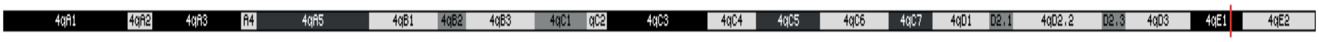

Filled site:

2 Poly_B2_3 (192 bp)

5′-TGACTCCCACTGTGCTATCAAAAATTAAAA**TA** [Poly_B2_3] A_{10} **TA**AGCCAGGCAGTGGTGACACATGCCTTTA-3′
3′-ACTGAGGGTGACACGATAGTTTTTAATTTT**AT** [Poly_B2_3] T_{10} **AT**TCGGTCCGTCACCACTGTGTACGGAAAT-5′

Empty site:

5′-GCCAAAAACTCTGACTCCCACTGTGCTATCAAAAATTAAAA**TA**AGCCAGGCAGTGGTGACACATGCCTTTAATC-3′
3′-CGGTTTTTGAGACTGAGGGTGACACGATAGTTTTTAATTTT**AT**TCGGTCCGTCACCACTGTGTACGGAAATTAG-5′

↑ 1st strand cleavage

SINE subfamily: B2

TSD: 2 bp
(Chr4, 146,493,190 - 146,493,204)

EN motif: 5′-TTTT/AT-3′

Poly_B2_4

chr9 (qD), +strand

Chr9 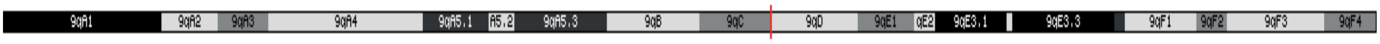

Filled site:

2 Poly_B2_4 (192 bp)

5′-GGTTCTAAAGTTTTTCGTTGTC**AAAAACTGCT** [Poly_B2_4] A_{10} **AAAAACTGCT**AAGAAATGTTAGTCTCATAT-3′
3′-CCAAGATTTCAAAAAGCAACAG**TTTTTGACGA** [Poly_B2_4] T_{10} **TTTTTGACGA**TTCTTTACAATCAGAGTATA-5′

Empty site:

5′-TCAAAAAAACAGAGGTTCTAAAGTTTTTCGTTGTC**AAAAACTGCT**AAGAAATGTTAGTCTCATATCTAGCTTACT-3′
3′-AGTTTTTTTGTCTCCAAGATTTCAAAAAGCAACAG**TTTTTGACGA**TTCTTTACAATCAGAGTATAGATCGAATGA-5′

↑ 1st strand cleavage

SINE subfamily: B2

TSD: 10 bp
(Chr9, 69,742,273 - 69,742,283)

EN motif: 5′-TTTT/GA-3′

Supplemental Figure S3. Structures of polymorphic retrotransposon insertions absent from the C57BL/6J reference genome (GRCm38/mm10).

For each structure, the relative location of the insertion is shown on the chromosome. The filled-site diagram denotes target-site duplications (TSDs), promoter monomer units for L1 insertions are shown as triangles, ORF1 and ORF2 are indicated as open rectangles. The truncation point for each insertion relative to the L1MdTf_I consensus(Sookdeo et al. 2013) is indicated. A_n/T_n indicates poly(A) sequence length. Below, the sequence of the empty site is shown, with TSDs and the site of first-strand cleavage indicated. Characteristics of each insertion (retroelement family, monomer number for L1 insertions, TSD length and chromosomal location, and endonuclease (EN) cleavage motif) are summarized below. 3' transductions are indicated as filled grey rectangles.
