# Supplementary material for: Heritable L1 retrotransposition in the mouse primordial germline and early embryo
Source: Genome Res. 2017 Aug;27(8):1395–405. doi: 10.1101/gr.219022.116 (PMC5538555; doi:10.1101/gr.219022.116)
Supplement: Supplemental Material [file supp_gr.219022.116_Supplemental_Table_4.pdf]

Supplemental Table 4: Target Site Characteristics of De Novo L1 Insertions

Insertion #	Target Site Genomic Location	Target-Site GC Content	
		**50 bp**	**20 kb**
1	intergenic; repeat (Lx10)	28%	46%
2	intergenic	26%	37%
3	intergenic	31%	37%
4	intergenic	26%	37%
5	intronic (*ano4*, antisense); repeat (L1mA5)	22%	38%
6	intergenic	28%	36%
7	intergenic; repeat (L1mus4)	43%	41%
8	intergenic	31%	37%
9	intergenic	29%	40%
10	intergenic	28%	35%
11	intergenic	33%	38%
	**average GC content:**	**30%**	**38%**

Supplemental Table 4. Target site characteristics of *de novo* L1 insertions. For each insertion, the target site type (intergenic, intronic, exonic) and whether the insertion occurred within an existing repeat sequence is indicated. The percent GC content of the insertion site, within a 50bp window and a 20kb window of the endonuclease cleavage site, is indicated.
